# Self-Organization of Minimal Anaphase Spindle Midzone Bundles

**DOI:** 10.1016/j.cub.2019.05.049

**Published:** 2019-07-08

**Authors:** Jonathon Hannabuss, Manuel Lera-Ramirez, Nicholas I. Cade, Franck J. Fourniol, François Nédélec, Thomas Surrey

**Affiliations:** 1 The Francis Crick Institute, 1 Midland Road, London NW1 1AT, UK; 2 Institut Curie, PSL Research University, CNRS, UMR 144, 75005 Paris, France; 3 London Research Institute, Cancer Research UK, 44 Lincoln’s Inn Fields, London WC2A 3LY, UK; 4 Sainsbury Laboratory, Cambridge University, Bateman Street, Cambridge CB2 1LR, UK

**Keywords:** mitotic spindle, anaphase, spindle midzone, microtubule, motor protein, kinesin, self-organization, *in vitro* reconstitution, computer simulation, Cytosim

## Abstract

In anaphase spindles, antiparallel microtubules associate to form tight midzone bundles, as required for functional spindle architecture and correct chromosome segregation. Several proteins selectively bind to these overlaps to control cytokinesis. How midzone bundles assemble is poorly understood. Here, using an *in vitro* reconstitution approach, we demonstrate that minimal midzone bundles can reliably self-organize in solution from dynamic microtubules, the microtubule crosslinker PRC1, and the motor protein KIF4A. The length of the central antiparallel overlaps in these microtubule bundles is similar to that observed in cells and is controlled by the PRC1/KIF4A ratio. Experiments and computer simulations demonstrate that minimal midzone bundle formation results from promoting antiparallel microtubule crosslinking, stopping microtubule plus-end dynamicity, and motor-driven midzone compaction and alignment. The robustness of this process suggests that a similar self-organization mechanism may contribute to the reorganization of the spindle architecture during the metaphase to anaphase transition in cells.

## Highlights

• Spindle midzone-like minimal microtubule bundles robustly self-organize *in vitro*
• PRC1 crosslinkers and KIF4A kinesins organize microtubules into well-defined bundles
• The PRC1/KIF4A ratio controls the extent of antiparallel microtubule overlap
• Kinesins organize microtubules by pulling mobile crosslinkers into full compaction

## Teaser

Hannabuss et al. identify a minimal set of proteins that can arrange microtubules *in vitro* into ordered antiparallel bundles, similar to the ones found in anaphase spindles. Using quantitative imaging and computer simulations, they outline a simple molecular mechanism that explains the self-organization phenomena and its robustness.

## Introduction

During mitosis, the microtubule cytoskeleton forms a bipolar spindle around chromosomes. When chromosomes are pulled toward the spindle poles during anaphase, spindle stability relies on microtubule bundles forming central antiparallel microtubule overlaps [1, 2]. These overlaps are a few micrometers long and align in the spindle midzone [3]. Correct central anaphase spindle formation is critical for successful chromosome segregation, correct positioning of the cleavage plane, and proper cytokinesis [4].

**Figure 1:**
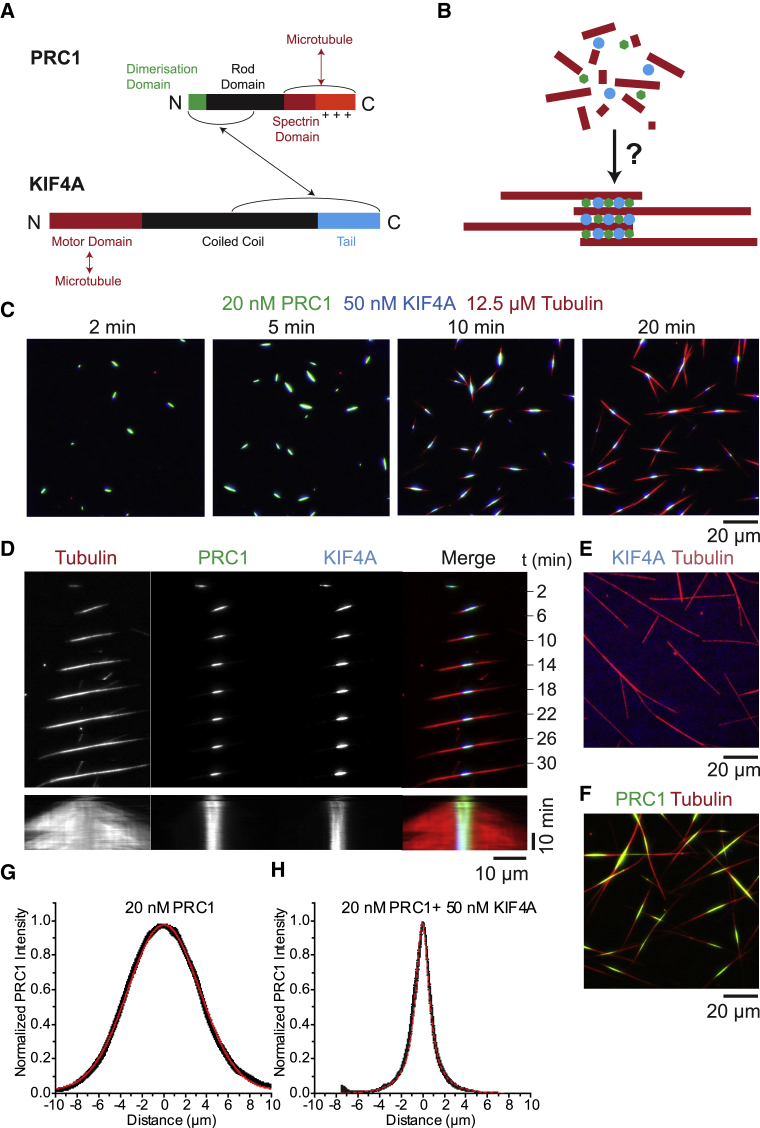
Self-Organization of Minimal Anaphase Midzone Bundles (A) Schematic of PRC1 (top) and KIF4A (bottom) domains and interactions. (B) Schematic of the formation of minimal anaphase midzone bundles. (C) Triple-color TIRF microscopy images showing the time course of self-organization of antiparallel microtubule bundles in the presence of 20-nM PRC1-Alexa546 (green), 50-nM KIF4A-mBFP (blue), and 12.5-μM Alexa647-tubulin (red). Times in minutes after initiating microtubule nucleation by a temperature shift to 30°C are shown. See Video S1. (D) Single- and triple-color TIRF microscopy image sequences (top) and kymographs (bottom) showing PRC1 and KIF4A accumulation in the central part of the antiparallel microtubule bundle; condition as in (C). (E) TIRF microscopy image of individual microtubules polymerized in a solution containing 50-nM KIF4A-mBFP (blue) and 12.5-μM Alexa647-tubulin (red), taken 20 min after initiating nucleation. (F) TIRF microscopy image of microtubule bundles polymerized in the presence of 20-nM PRC1-Alexa546 (green) and 12.5-μM Alexa647-tubulin (red), taken 20 min after initiating nucleation. See Video S2. (G and H) Average normalized PRC1 fluorescence intensity profiles along the bundle axis at 30 min, for microtubule bundles formed in the presence of 20-nM PRC1 only (G; n = 9) and with 20-nM PRC1 and 50-nM KIF4A (H; n = 14). The linewidth shows the SE of the average. The profiles have been fit (red line) with a Gaussian (G; σ = 6.6 μm) and Lorentzian distribution (H; σ = 1.5 μm), respectively. The temperature was 30°C. See also Figures S1 and S6.

Several proteins localize to the central anaphase spindle, contributing to its organization [1, 2, 4]. A critical player is PRC1 (protein required for cytokinesis 1) [5, 6, 7, 8], which is conserved within metazoans, plants, and yeast [9, 10, 11, 12, 13]. It preferentially crosslinks antiparallel microtubules and recruits other anaphase spindle proteins [14, 15, 16, 17]. PRC1 is a homodimer and binds microtubules with its spectrin domains and neighboring unstructured positively charged regions, keeping antiparallel microtubules separated by ∼35 nm (compared to an outer microtubule diameter of 25 nm; Figure 1A) [14, 17, 18, 19].

In human cells, one of the proteins recruited by PRC1 is the kinesin-4 KIF4A, a plus-end-directed motor that is conserved in metazoans [6, 8, 20, 21, 22, 23]. KIF4A is a homodimer with its N-terminal motor domain followed by a long coiled coil region and a C-terminal tail domain, being 116 nm long [24]. The C-terminal part of KIF4A interacts with the N-terminal dimerization domain of PRC1 located in the center of the molecule (Figure 1A) [6, 8, 20, 21, 22, 23]. KIF4A limits the length of central antiparallel microtubule overlaps in the anaphase spindle [6, 8, 25], as kinesin-4 motors can inhibit microtubule growth [14, 26, 27].

Other proteins also play important roles in central anaphase spindle function, including regulators, such as kinases and phosphatases, that modulate binding affinities of PRC1 and KIF4A in a cell-cycle-dependent manner [6, 8, 25]. However, *in vitro* experiments with purified proteins have shown that PRC1 and kinesin-4 together are sufficient to promote the formation of antiparallel microtubule overlaps with controlled length between microtubule pairs [14, 28].

*Xenopus laevis* PRC1 and kinesin-4 were demonstrated to be sufficient to produce stable antiparallel overlaps between pairs of immobilized microtubules that grew dynamically toward each other and to control the overlap length [14]. PRC1 localized selectively to overlaps and recruited *Xenopus* kinesin-4 that inhibited microtubule plus-end growth. Kinesin-4 did not appreciably transport PRC1 under conditions of high protein binding and unbinding turnover (high ionic strength) [14]. Overlap length was controlled by overlap length-dependent inhibition of microtubule growth.

In a different experiment using human proteins under conditions of higher binding affinities (lower ionic strength), KIF4A transported PRC1 along single immobilized static microtubules, leading to accumulation of both proteins at microtubule plus ends [29]. When stabilized microtubules were added, antiparallel microtubule transport was observed [28]. Antiparallel sliding slowed down and eventually stopped, presumably due to steric hindrance, leading to the formation of short overlaps between pairs of stabilized microtubules. Overlap length depended on the initial length of the overlaps formed by random collision of two microtubules [28].

Different mechanisms appear to explain antiparallel microtubule overlap formation by PRC1 and kinesin-4, depending on the particular *in vitro* conditions. As previous studies involved microtubule immobilization or stabilization, it remains unclear whether anaphase spindle-like microtubule overlaps can also form without such mechanical or biochemical constraints.

Here, we demonstrate that, over a wide range of conditions, human PRC1 and KIF4A robustly organize freely nucleating microtubules into minimal anaphase-like midzone bundles. We identify the ratio of PRC1 to KIF4A as a critical control parameter determining antiparallel microtubule overlap length in these bundles. These experiments, together with computer simulations, show that selective recruitment to overlaps, inhibition of microtubule plus-end growth, antiparallel microtubule sliding, and protein compaction work together to establish a stable overlap. Thus, PRC1 and kinesin-4 are sufficient to robustly form minimal central anaphase spindle-like microtubule arrays from an initially homogeneous solution.

## Results

### Self-Organization of Minimal Central Anaphase Spindle Structures

We mixed fluorescently labeled human PRC1, KIF4A, and tubulin at a medium ionic strength, where PRC1 promotes sufficient microtubule polymerization in the range of physiological PRC1 concentrations (∼10–100 nM; Figures 1B and S1A–S1C), confirming a property of PRC1 that had been noted earlier [19]. Using total internal reflection fluorescence (TIRF) microscopy, we observed short microtubule bundles forming in solution within minutes, with PRC1 and KIF4A bound (Figure 1C; Video S1). Microtubule ends then grew outward from the short regions of PRC1 and KIF4A accumulation, forming flanking parallel extensions, as indicated by the absence of PRC1. Overall, these bundles were symmetric with a well-focused central antiparallel microtubule overlap as indicated by the accumulation of PRC1 there (Figures 1C and 1D).

Video S1. Self-Organization of Minimal Midzone Bundles in the Presence of PRC1 and KIF4A, Related to Figure 1 Conditions as in Figure 1C: 20 nM PRC1-Alexa546 (green), 50 nM KIF4A-mBFP (blue) and 12.5 μM Alexa647-tubulin (red). Times in minutes after initiating microtubule nucleation by a temperature shift to 30°C.

KIF4A alone did not significantly bind or bundle microtubules, confirming that this motor is not in itself an efficient microtubule crosslinker [14, 28] (Figure 1E). *In vitro* and in cells, PRC1 is required to recruit kinesin-4 to antiparallel microtubules [6, 14, 28]. In contrast, PRC1 alone bundled microtubules as expected (Figures 1F and S1D; Video S2). These bundles had considerably less focused central overlaps than bundles with both PRC1 and KIF4A present, as average PRC1 intensity profiles along the bundle axis demonstrate (Figures 1G and 1H). PRC1-only bundles also had longer flanking parallel extensions than PRC1/KIF4A bundles.

Video S2. Bundle Formation in the Presence of PRC1, Related to Figure 1 Conditions as in Figure 1F: 20 nM PRC1-Alexa546 (green) and 12.5 μM Alexa647-tubulin (red). Times in minutes after initiating microtubule nucleation by a temperature shift to 30°C.

**Figure 2:**
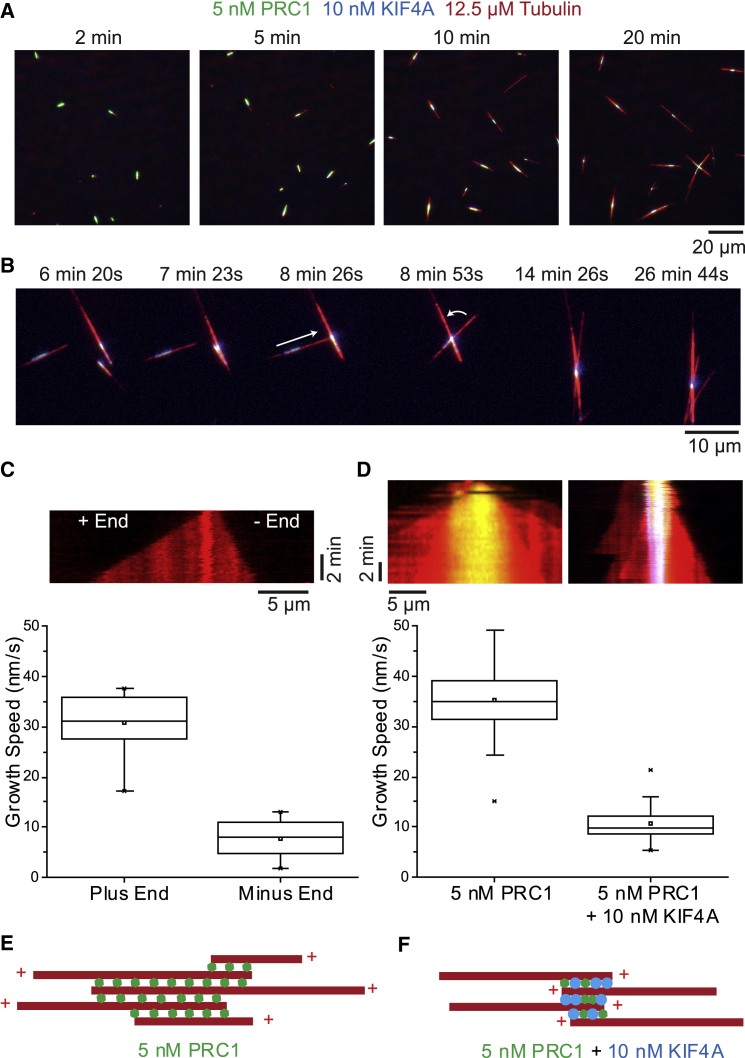
Microtubule Organization in Minimal Central Anaphase Spindles (A) TIRF microscopy images of self-organization of minimal anaphase midzone bundles in the presence of 5-nM PRC1-Alexa546 (green), 10-nM KIF4A-mBFP (blue), and 12.5-μM Alexa647-tubulin (red). Times in minutes after initiating microtubule nucleation by a temperature shift to 30°C are shown. (B) Image sequence showing an example of antiparallel midzone bundle fusion and alignment; condition as in (A). (C) (Top) Kymograph of a representative microtubule growing in the presence of 50-nM KIF4A-mBFP only, as in Figure 1E. (Bottom) Boxplot shows the speeds of plus and minus ends from individual dynamic microtubules (n = 18). The box represents the interquartile range (IQR) and whiskers represent the range (-outliers); x = outliers (>1.5 × IQR); box line, median; square, mean. (D) Kymographs (top) and corresponding boxplots (bottom) showing the growth speeds of microtubules growing outward in minimal midzone bundles assembled under conditions as in (A) (n = 32) and from bundles formed only in the presence of 5-nM PRC1-Alexa546 (n = 57). (E) Schematic illustrating the microtubule orientations in PRC1-only bundles (as in Figure 1F). (F) The inverted microtubule orientation in antiparallel bundles formed by both PRC1 and KIF4A (as in A and B and Figures 1C and 1D).

PRC1 and KIF4A reliably formed antiparallel bundles with focused overlaps over a range of concentrations (Figure 2A shows an example at lower PRC1 and KIF4A concentrations). When bundles contacted each other, they fused and aligned driven by plus-end-directed KIF4A motility (Figure 2B). This suggests that microtubule plus-end segments form the central antiparallel overlaps in the presence of PRC1 and KIF4A and that parallel microtubules point outward with their minus ends. Indeed, outward growing microtubules grew with the typical minus-end growth speed (Figures 2C and 2D) and remained dynamic (Figure 1D). In contrast, plus ends stopped growing during overlap formation, most likely due to the action of accumulated KIF4A. Kinesin-4 inhibits microtubule plus-end growth, in contrast to PRC1 that does not affect growth speed [14].

Without KIF4A, fusion events moving overlaps together were not observed. For PRC1-only bundles, the speed of outward microtubule growth corresponded to the typical plus-end growth speed at the tubulin concentration used (Figures 2C and 2D). This indicates a central random orientation of microtubules in PRC1-only bundles and a parallel microtubule orientation in extensions beyond the PRC1 region; there, plus ends point outward as a consequence of faster plus- than minus-end growth. Bundles formed by PRC1 alone have a different architecture than bundles formed by PRC1 and KIF4A together (Figures 2E and 2F).

Hence, PRC1 and KIF4A are necessary and sufficient to organize dynamic microtubules into antiparallel bundles with an architecture resembling the organization of microtubules in the central anaphase spindle. Compared to previous *in vitro* reconstitutions with PRC1 and kinesin-4, here, all microtubules are initially free to diffuse (no surface immobilization) and essentially all microtubules become incorporated into minimal midzone bundles by an efficient self-organization process.

### Time Course of Minimal Anaphase Microtubule Overlap Formation

**Figure 3:**
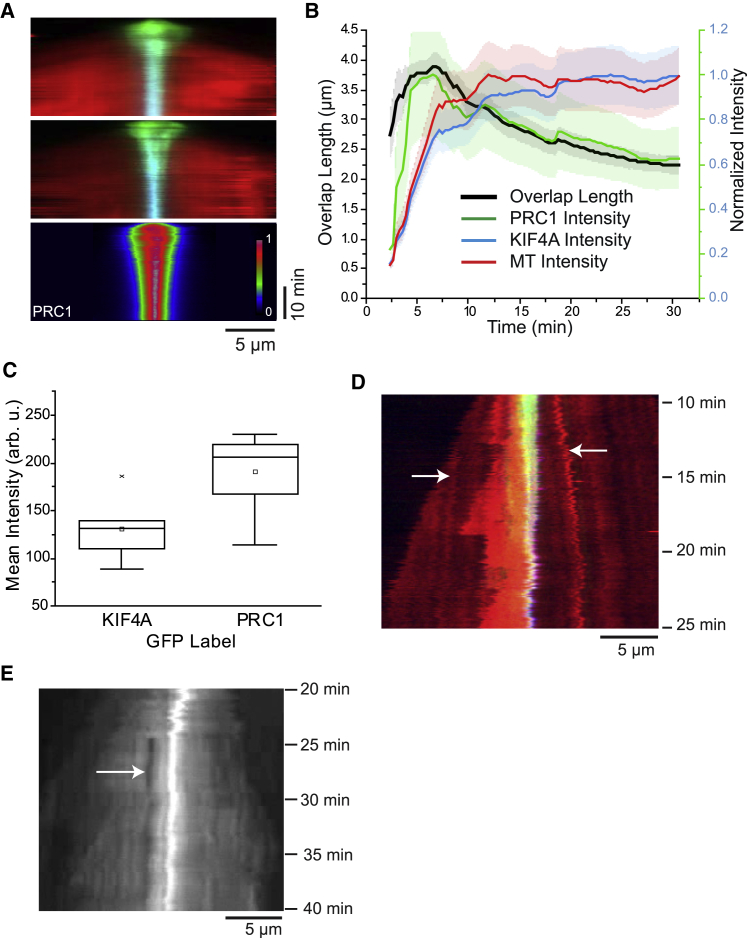
Time Course of Minimal Midzone Bundle Formation (A) (Top) Kymographs showing the time course of the formation of two antiparallel bundles in the presence of 20-nM PRC1-Alexa546 (green), 50-nM KIF4A-mBFP (blue), and 12.5-μM Alexa647-tubulin (red), imaged by TIRF microscopy (condition as in Figures 1C and 1D). (Bottom) Average normalized PRC1 intensity (n = 14) is shown. (B) Mean overlap length and normalized mean total fluorescence intensity measured in the overlap region of minimal midzone bundles for Alexa647-tubulin, PRC1-Alexa546, and KIF4A-mBFP plotted as a function of time (n = 17); protein concentrations as in (A). The shaded areas show the SE. (C) Boxplots showing the mean intensity of KIF4A and PRC1 in the overlap region at the endpoint of overlap formation (∼30 min; n = 8); protein concentrations as in (A). To enable a comparison of absolute final amounts of the proteins, two sets of experiments were done with either the KIF4A or PRC1 labeled with GFP, showing a 1.5- to 2-fold excess of PRC1 over KIF4A. (D) Kymograph showing the tubulin fluorescence of an antiparallel bundle consisting of 3 microtubules soon after nucleation. The white arrows indicate speckles of higher tubulin labeling density, which show slow (∼3 nm/s) antiparallel sliding. Time is in minutes after initiating microtubule nucleation. Protein concentrations are as in Figure 2A. (E) Kymograph showing the tubulin fluorescence of a minimal midzone bundle formed in the presence of 5-nM PRC1-Alexa546, 50-nM KIF4A-mBFP, and 12.5-μM Alexa647-tubulin. A bleach mark was placed on the bundle outside of the central antiparallel microtubule overlap ∼25 min after initiating nucleation. The distance between the bleach mark and the overlap remained constant. The temperature was 30°C. See also Figures S2 and S3.

Kymographs of minimal anaphase midzone bundles show that PRC1 and KIF4A-rich antiparallel microtubule overlaps first elongate, reaching a peak length, and then slowly shrink toward a stable length as dynamic microtubule minus-end growth elongates the bundles (Figure 3A). We extracted from our videos the length of the central overlap regions and the total fluorescence intensities of PRC1, KIF4A, and tubulin in the overlaps (Figure S2). The average overlap length reached a peak of 3.8 μm ∼7 min after nucleation started and then decreased slowly over time, finally approaching a length of 2.4 μm after ∼30 min (Figure 3B, black line).

The total amount of microtubule polymer in the overlap plateaued, as indicated by the normalized average tubulin intensity measured in the overlap. Thus, some microtubules are incorporated into the overlap while its length slowly decreases (see Discussion). In contrast, the total PRC1 amount in the overlap followed a trend similar to overlap length and not tubulin amount (Figure 3B, green line); this suggests that part of the PRC1 leaves the overlap, possibly binding to weak binding sites on the long parallel microtubule extensions outside the overlap.

The total KIF4A amount in the overlap increased slowly over the duration of the experiment (Figure 3B, blue line) following the trend of the total tubulin amount. We compared the absolute final amounts of GFP versions of PRC1 and KIF4A in antiparallel overlaps at the end of overlap formation (Figure 3C): this demonstrated that PRC1 was in excess over KIF4A. This agrees with PRC1 recruiting KIF4A under our conditions (compare Figures 1C and 1E), as observed also previously in antiparallel microtubule pairs [14]. Fluorescence recovery after photobleaching (FRAP) experiments at the end of minimal midzone bundle formation showed that PRC1 displayed no detectable recovery (Figures S3A and S3B), indicating a strong affinity. KIF4A showed slow partial turnover (Figures S3C and S3D), suggesting some unbinding and rebinding.

Slow antiparallel overlap shortening could be due to KIF4A-mediated sliding of microtubules until a final state is reached. Indeed, sliding was directly observed in bundles consisting of only a few microtubules: microtubule speckles were occasionally evident and moved outward in concert with overlap shortening (Figure 3D). This agrees with previous observations of antiparallel microtubule sliding in pairs of stabilized microtubules in the presence of PRC1 and KIF4A [28]. Sliding eventually stopped, as revealed by bleaching a mark outside of the overlap region after minimal midzone bundle formation; the distance between the bleach mark and the center of the bundle remained constant (Figure 3E).

The final overlap length was considerably longer than the local accumulation of PRC1 and KIF4A on single stabilized microtubules, previously called “end tags” [28, 29] (Figure S3E), suggesting that the mechanism determining overlap length is distinct from that governing end accumulation on individual microtubules under the conditions studied here.

### The PRC1/KIF4A Ratio Controls Overlap Length

**Figure 4:**
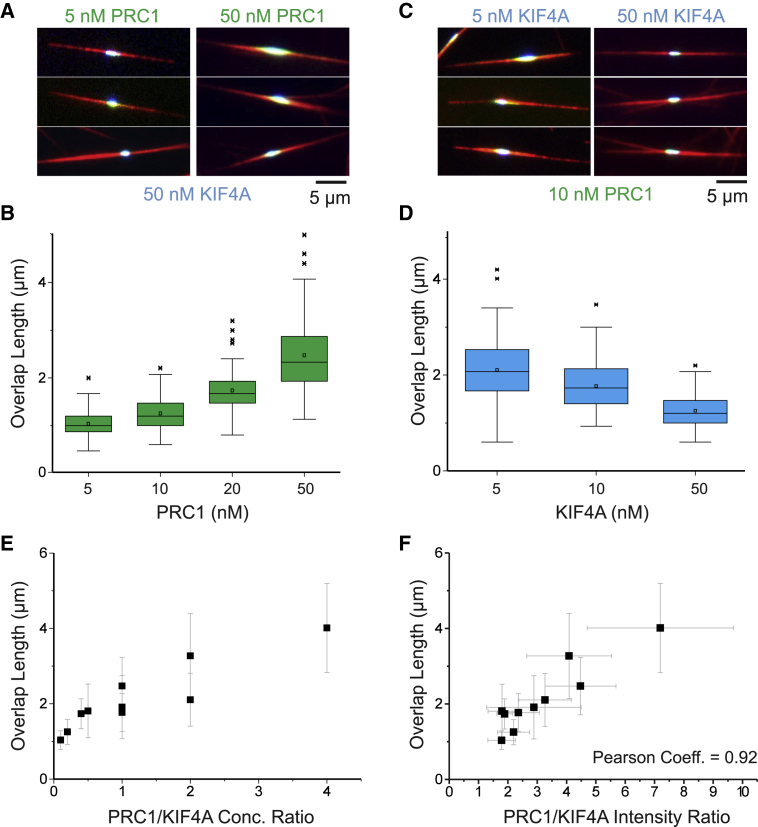
PRC1 and KIF4A Control Final Antiparallel Microtubule Overlap Length (A) Triple-color TIRF microscopy images showing minimal midzone bundles at different PRC1-Alexa546 concentrations (5 nM and 50 nM, green), with the same KIF4A-mBFP concentration (50 nM, blue), taken ∼40 min after initiating nucleation. See Video S3. (B) Boxplot showing the distribution of final overlap lengths in self-organized minimal midzone bundles measured at *t* = ∼40 min in the presence of 50-nM KIF4A-mBFP and varying PRC1-Alexa546 concentrations as indicated; n > 100 overlaps for each condition. (C) Triple-color TIRF microscopy images showing minimal midzone bundles at different KIF4A-mBFP concentrations (5 nM and 50 nM, blue), with the same PRC1-Alexa546 concentrations (10 nM, green), taken ∼40 min after initiating nucleation. See Video S4. (D) Boxplot showing the distribution of final overlap lengths in the presence of 10-nM PRC1-Alexa546 and varying concentrations of KIF4A-mBFP. Box represents IQR and whiskers represent range (-outliers); x = outliers (>1.5 × IQR); box line, median; square, mean. (E) Scatterplot of the mean final overlap length as a function of the PRC1/KIF4A concentration ratio. Overlap lengths were measured for the PRC1/KIF4A concentration pairs (in nM/nM): 5/5, 5/10, 5/50, 10/5, 10/10, 10/50, 20/5, 20/10, 20/50, and 50/50 (n > 93 overlaps per condition); error bars represent SD. (F) Scatterplot of the mean final overlap length as a function of the mean total PRC1/KIF4A fluorescence intensity ratio as measured in the same overlaps as in (E); error bars represent SD. The Alexa647-tubulin concentration was always 12.5 μM. The temperature was 30°C. See also Figures S3 and S4.

Varying the PRC1 and KIF4A concentrations revealed that higher PRC1 concentrations led to longer final overlaps (Figures 4A and 4B; Video S3), whereas higher KIF4A concentrations shortened them (Figures 4C and 4D; Video S4). However, changing concentrations had little effect on the total number of microtubules in the bundles, as estimated from the tubulin fluorescence intensity (Figures S4A and S4B), demonstrating a selective effect on overlap length. More precisely, overlap length increased with an increasing ratio of PRC1 to KIF4A concentration (Figure 4E). The strongest correlation exists between overlap length and the ratio of the PRC1 and KIF4A amounts measured directly in the antiparallel microtubule overlaps (Figures 4F and S4B). Overlap length appears to depend linearly on this control parameter.

Video S3. Minimal Midzone Bundle Formation in the Presence of KIF4A and Two Different PRC1 Concentrations, Related to Figure 4 Conditions as in Figure 4A: 5 nM and 50 nM PRC1-Alexa546 (green), 50 nM KIF4A-mBFP (blue) and 12.5 μM Alexa647-tubulin (red). Times in minutes after initiating microtubule nucleation by a temperature shift to 30°C.

Video S4. Minimal Midzone Bundle Formation in the Presence of PRC1 and Two Different KIF4A Concentrations, Related to Figure 4 Conditions as in Figure 4C: 10 nM PRC1-Alexa546 (green), 5 nM and 50 nM KIF4A-mBFP (blue) and 12.5 μM Alexa647-tubulin (red). Times in minutes after initiating microtubule nucleation by a temperature shift to 30°C.

**Figure 5:**
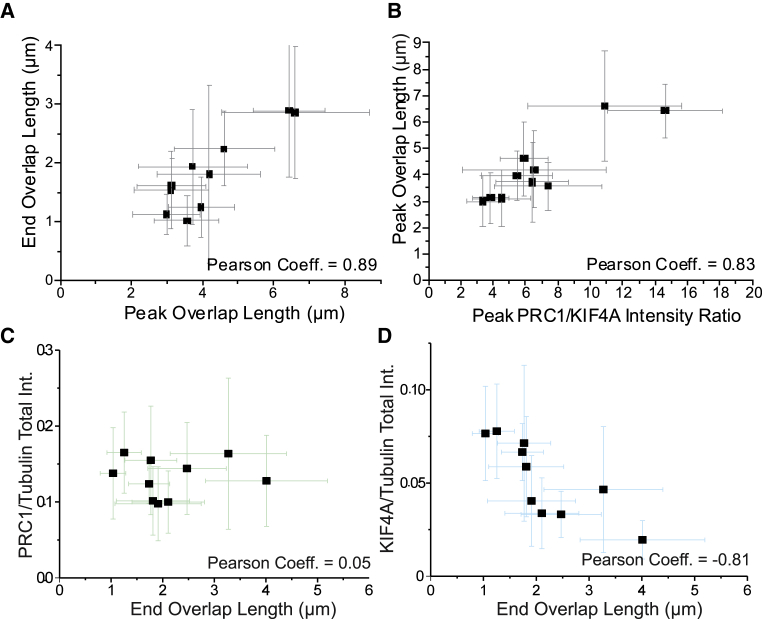
Overlap Length Correlations (A) The maximum (peak) lengths and the final lengths of antiparallel microtubule overlaps in self-organized minimal midzone bundles were extracted from time courses of overlap length. For each combination of PRC1 and KIF4A concentrations (datasets as used for Figures 4C and 4D), the mean peak overlap length and mean final overlap length show a positive correlation. (B) Plot of the mean peak overlap length against the mean PRC1/KIF4A intensity ratio measured in the overlap at the time of its maximum length, also demonstrating a positive correlation. Errors are SD. (C and D) Mean PRC1/tubulin (C) and mean KIF4A/tubulin (D) total fluorescence intensity ratios in the overlap region versus the mean end overlap length, calculated from the same datasets as used for (A) and (B). See also Figure S4.

The peak (maximum) overlap length also correlated well with the final overlap length (Figure 5A) and was again set by the ratio of PRC1 to KIF4A amount in the overlap, this time measured at the time of maximum peak length (Figure 5B); this indicates that the PRC1/KIF4A ratio controls overlap length throughout the time course of minimal anaphase midzone bundle formation.

Lastly, we measured the final PRC1 and KIF4A densities in overlaps, i.e., the ratio of their fluorescence intensity to the tubulin fluorescence intensity (proportional to the PRC1 and KIF4A amounts per tubulin amount in the overlaps). Remarkably, no clear dependence of the final PRC1 density on the end overlap length was detected (Figure 5C), but the final KIF4A density was clearly reduced in longer overlaps (Figure 5D).

In summary, PRC1 controls the microtubule mass, initiates antiparallel microtubule bundle formation, and recruits KIF4A. KIF4A then sets the peak overlap length by stopping microtubule growth depending on the KIF4A concentration and hence its density in the overlap, as previously observed for immobilized microtubule pairs [14]. Final overlap length is then the result of an adjustment process caused by the redistribution of some PRC1 molecules as minus ends grow out, forming the finished self-organized minimal midzone bundles.

### Computational Model

To analyze the mechanism of microtubule overlap formation, we created a model using Cytosim [30]. Where possible, we used measured parameter values (Table S1). Key features of the model are PRC1-KIF4A association, discrete binding sites on microtubules, steric hindrance as lattice sites become saturated, and different lattices for PRC1 and KIF4A. For simplicity, we consider only a pair of antiparallel microtubules of constant length (5 μm).

PRC1 crosslinks and diffuses on microtubules: its two heads independently bind to an 8-nm lattice on different microtubules and move by stochastically stepping to neighboring sites in a force-dependent manner. A lattice site is limited to one PRC1 head, and PRC1 cannot step out of the microtubule at the plus or minus end. With one head bound, motion is diffusive and unbiased (0.1 μm^2^/s) [31]. With two heads bound, PRC1 is modeled as an elastic linker. Force is exerted on the microtubules, proportionally to the distance between the heads.

**Figure 6:**
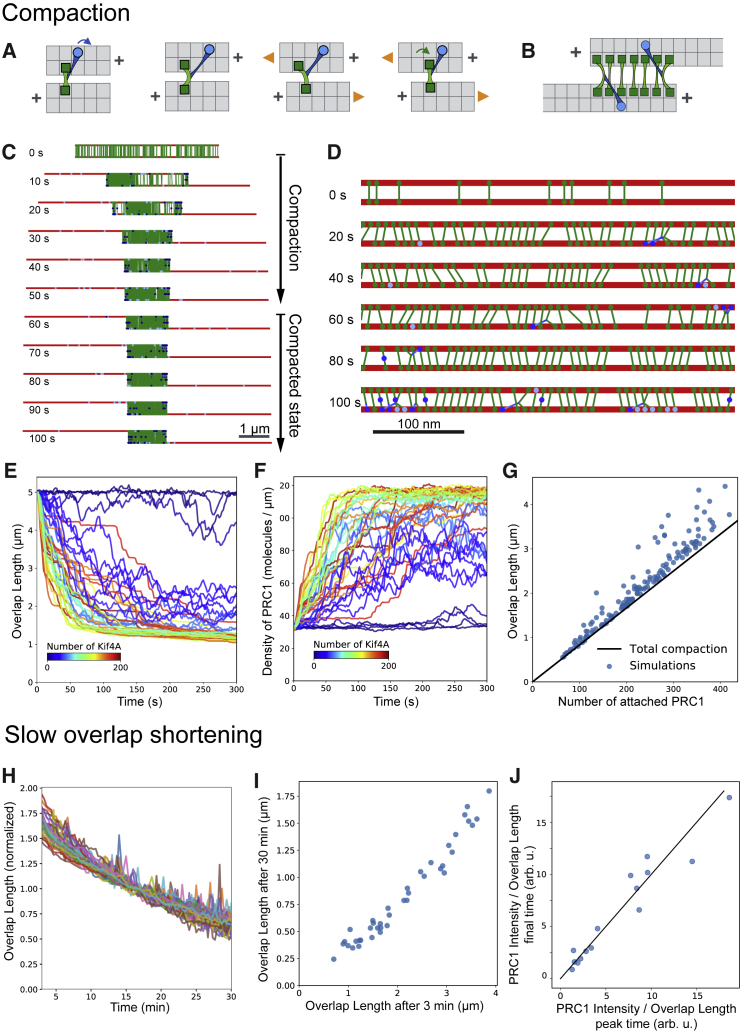
Model of Overlap Formation (A) Sliding mechanism. KIF4A pulls a PRC1 molecule that is linking two microtubules, inducing strain on both PRC1 heads. The top PRC1 head releases the strain by biased diffusion toward the plus end. The bottom PRC1 head releases the strain by biased diffusion or microtubule sliding. (B) PRC1 compaction stalls microtubule sliding. (C) Representative simulation with 2 microtubules (length 5 μm), 200 PRC1 and 132 KIF4A (Table S1, set 1). See Video S5. (D) Magnified view of the simulation shown in (C). See Video S6. (E) Dynamics of overlap length in simulations (mircotubule length 5 μm; 200 PRC1; Table S1, set 1). Lines stand for individual simulations where the number of KIF4A varies from 0 to 200 (see color scale). (F) PRC1 density for the same simulations as in (E). (G) Scatterplot showing the correlation between the length of the overlap and the number of PRC1 molecules attached in the overlap after 300 s. Each dot represents one simulation (Table S1, set 1) but with randomized numbers of KIF4A (30–200) and PRC1 (100–600) and microtubule length (3–7 μm). The diagonal black line represents full compaction (i.e., one molecule per 8 nm). (H) Shortening of overlaps in different simulations containing 100 KIF4A and between 100 and 600 PRC1 (Table S1, set 1). In these simulations, compaction is reached earlier than 3 min after KIF4A addition (E), and this plot focuses on later times. (I) Overlap at 3 min (when total compaction reached) versus overlap at 30 min for data shown in (H). (J) Comparison between the experimental ratio between PRC1 intensity and overlap length, at the time of maximal overlap (x axis) and at final time (y axis). Dots represent individual overlaps from the data shown in Figure 3B. The diagonal indicates perfect conservation of this quantity. See also Figure S5.

The KIF4A motor binds to an 8-nm lattice that is distinct from the PRC1 lattice (Figures 6A and S5A). This reflects KIF4A’s expected ability to reach a different microtubule protofilament when associated with PRC1, due to its long length [24]. Supporting this, KIF4A can move in microtubule overlaps where the PRC1 density is so high that individual PRC1 molecules do not diffuse [14]. KIF4A steps stochastically toward the plus end of the microtubule in a force-dependent manner. The unhindered KIF4A speed is 800 nm/s [14], and KIF4A remains attached upon reaching the microtubule plus end [29].

KIF4A can associate with PRC1 (Figures S5A–S5C): their association is represented by an elastic link joining KIF4A to the middle of the PRC1 molecule [29] (Figure 6A). On a single microtubule, KIF4A transports PRC1; in a microtubule pair with PRC1 being attached to both microtubules, the tension created by motor movement is dissipated either through diffusion of PRC1 or microtubule sliding (Figure 6A). Although not every kinesin step leads to microtubule displacement (Figure S5D), antiparallel sliding is a characteristic property of the KIF4A/PRC1 crosslink. Importantly, the movement of a PRC1 molecule can be blocked by neighboring PRC1 molecules. As PRC1 diffuses relatively fast compared to the movement of KIF4A, such obstacles are likely to diffuse away, unless a “traffic jam” of PRC1 forms, in which case KIF4A movement is hindered as long as it is bound to PRC1 (Figure 6B).

For a complete list of model assumptions, see the Computer Simulations section in STAR Methods.

### Fast PRC1 Compaction as Microtubules Slide Apart

For a wide range of motor and PRC1 numbers and initial overlap lengths, microtubules initially slide fast and then slow down to eventually form long-lasting antiparallel overlaps (Figures 6C and 6D; Videos S5 and S6). As overlap length decreases (Figure 6E), the PRC1 density increases (Figure 6F), eventually reaching full compaction, where almost every PRC1 binding site in the overlap is occupied and the length is set by the total number of PRC1 molecules (Figures 6B and 6G). Sliding is driven by PRC1-KIF4A complexes connecting two microtubules (Figure 6D). These results are consistent with previous experiments with PRC1 and KIF4A in microtubule pairs, where the microtubule sliding speed was fast until steric hindrance inhibited sliding within 1 or 2 min [28].

Video S5. Simulation of Sliding-Driven PRC1 Compaction in an Antiparallel Microtubule Overlap, Related to Figure 6 Parameters as in Figure 6C. Microtubules (red) are crosslinked by PRC1 (green). KIF4A can be simultaneously attached to PRC1 and a microtubule (dark blue) or only bound to a microtubule (light blue). Unbound KIF4A molecules are not shown. Microtubule length is 5 μm. Total time of the video is 300 s (as indicated by the timestamp).

Video S6. Simulation of Sliding-Driven PRC1 Compaction in an Antiparallel Microtubule Overlap—Magnified, Related to Figure 6 Higher magnification of the central part of the simulated overlap shown in Video S5 played at a slower rate, from simulated time 5 s to 11 s (as indicated by the timestamp), to illustrate the sliding mechanism, particularly the interaction between KIF4A and PRC1. Color code as in Video S5. To visualize the microtubule sliding, every 8th lattice site (every 64 nm) was blackened.

In the low-density regime, an analytical expression can be obtained (STAR Methods), showing that the sliding speed is set by the speed of the motor and by the ratio between the drag of the microtubules against the fluid and the drag associated with diffusible crosslinkers. This relationship expresses how much of the KIF4A work is used to slide the microtubules, instead of being dissipated by dragging PRC1 along the microtubule. Because the fluid drag is small *in vitro*, the microtubules could in principle slide at the unloaded speed of KIF4A. However, when the overlap is crowded, jamming becomes the main limiting factor for sliding. Total compaction is reached for a wide range of values of motor number, binding and unbinding turnover kinetics (parameter set 1 versus 2 in Table S1), and motor force (parameter set 1 versus 4 in Table S1) whenever the density of PRC1-KIF4A complexes is higher than ∼10/μm (Figure S5E). Because experimentally the PRC1 density in final overlaps is roughly the same across all conditions (Figure 5C), the number of motors is probably always sufficient to reach compaction. This also happens when KIF4A is allowed to fall off at microtubule plus ends (Figure S5E; parameter set 3 in Table S1). In contrast, if PRC1 is allowed to fall off the microtubule ends by diffusion, microtubules slide completely apart (data not shown).

The model thus recapitulates the formation of long-lasting microtubule overlaps in which the PRC1 density is kept constant by the action of KIF4A, creating a state close to “total compaction.” Most likely, this compaction happens in our experiments early on, during the period where PRC1 intensity increases faster than microtubule intensity, which occurs while overlaps are still extending. Later, the PRC1 intensity roughly follows overlap length (Figure 3B). This model illustrates how KIF4A together with PRC1 can drive the relative sliding of antiparallel microtubules in a manner that will automatically stop before the overlap vanishes, as PRC1 jams when the system reaches full compaction.

### Slow Shortening of Overlap Length Is Mediated by PRC1 Unbinding from the Overlap

In our experiments, the overlap length slowly decreased with similar dynamics as some of the PRC1 dissociated (Figure 3B). The shrinkage speed was 100 times slower than the unloaded motor speed. Given that, in our model, KIF4A sliding keeps PRC1 at near-total compaction, a slow loss of PRC1 leads to a decrease of the overlap length (Figures 6H and S5F). For a constant off rate per PRC1 molecule, longer overlaps lose more PRC1 per unit time than shorter overlaps, causing shortening to slow down over time. Thus, a correlation between initial and final overlap length is obtained in simulations (Figure 6I), similar to the experimentally observed correlation between peak and final overlap length (Figure 5A). If compaction is reached, one would expect the ratio of PRC1 intensity over overlap length to remain constant, and this is indeed experimentally observed if one compares peak and final time points (Figure 6J).

## Discussion

We have shown that PRC1 and KIF4A are sufficient to organize dynamic microtubules into bundles with a defined central antiparallel microtubule overlap. Self-organization proceeds from homogeneous solution, relying on the combination of several activities: PRC1 promotes microtubule formation; bundles antiparallel microtubules; and recruits KIF4A to overlaps. KIF4A blocks microtubule growth in an overlap length-dependent manner and, together with PRC1, slides antiparallel microtubules until full compaction of PRC1 is reached. This self-organization process combines elements previously observed separately in experiments with microtubule pairs [14, 28].

Our simulations explain why KIF4A bound to PRC1 slides antiparallel microtubules (Figures 6A and S5A) and why sliding stalls due to crowding as PRC1 compacts (Figures 6B–6G). PRC1 jamming can also explain why previously observed KIF4A/PRC1-mediated microtubule pair sliding stalled within 1 or 2 min [28]. This time is similar to the time needed to reach PRC1 compaction in our simulations and peak overlap length in our self-organization experiments. In previous sliding experiments with stable microtubules, overlap length depended on random initial overlap length [28]; however, here we find that peak overlap length is controlled by KIF4A, as observed previously with immobilized dynamic microtubules [14].

During the later phase of bundle self-organization, overlaps shorten very slowly (∼30 min) following the kinetics of PRC1 slowly dissociating from the overlap (Figure 3B). This phase is different from the fast compaction phase during which protein densities increase until sliding stalls [28]. In our model, during slow sliding, motor forces keep PRC1 at total compaction, preventing its rebinding, in agreement with our FRAP data (Figures S3A and S3B).

This mechanism explains (1) why slow overlap shortening and PRC1 unbinding happen with the same kinetics (Figures 3B and 6H), (2) why peak and end overlap length are proportional (Figures 5A and 6I), and (3) why the PRC1 density in final overlaps is independent of overlap length (Figures 5C and 6G). Importantly, as final overlap length is proportional to intermediate peak length, the ratio of PRC1/KIF4A that controls peak length also controls final overlap length.

Entropic forces generated by Ase1 molecules in antiparallel microtubule overlaps can stall motor-driven antiparallel microtubule sliding by kinesin-14 [31, 32]. However, for the PRC1/KIF4A system, such a force-balance model does not easily explain why the final PRC1 density in overlaps is independent of their length or the KIF4A density [33].

We have not attempted to model here microtubule growth, the growth inhibiting effect of KIF4A [14, 26], or the stimulating effect of PRC1 on microtubule formation [19], because their molecular mechanisms are unknown. As PRC1 does not bind efficiently to single microtubules and does not affect the growth speed of microtubules [14], it may stabilize microtubules by inhibiting microtubule shrinkage or promoting rescues particularly in bundled microtubules. Importantly, because the number of microtubule bundles increased with the PRC1 concentration (Figure S1B), but the number of microtubules per bundle was roughly unaffected (Figure S4A), changing the PRC1 concentration affects overlap length indirectly by changing the KIF4A density in overlaps. Therefore, mechanistically, it is KIF4A that controls overlap length in our experiments.

Our model with only two microtubules does not explain why the microtubule number in self-organized bundles seems to increase over time without changing the PRC1 compaction state (Figure 3B). Possibly, the PRC1 density is higher in the inner part of the bundles (comprising on average ∼18 microtubules), which eventually may determine bundle behavior. Similarly, the KIF4A density may not be constant throughout the bundles, as it binds more slowly than PRC1. Higher resolution imaging will be required to better understand these morphological characteristics of the minimal midzone-like bundles, including the degree of microtubule plus-end alignment.

In our simulations, the PRC1 dissociation rate was assumed to be constant, but it seems to slow down in the experiments, because sliding eventually stops almost completely (Figure 3B). Nevertheless, the kinetics of overlap shortening and PRC1 unbinding are very similar, in agreement with the basic feature of our model. A reduction of the dissociation rate in the experiments may be due to PRC1 becoming trapped in overlaps of bundles consisting of many microtubules, a situation quite different from the simulated microtubule pair. Interestingly, during anaphase B in fission yeast, Ase1 turnover is reduced at the midzone [9], suggesting an important functional role for PRC1/Ase1 turnover regulation in cells. Measuring PRC1 turnover also in other species will be important.

Feedbacks in the mechanism of minimal midzone overlap formation may allow the cell to regulate protein activities without losing overlap integrity. If additional forces extend the spindle in anaphase B, reducing overlap length, microtubules would be expected to grow again in response to a decreasing amount of bound KIF4A in the overlap [14], thus preventing the collapse of the antiparallel connection, which is a landmark of anaphase B across organisms [34]. However, growth cannot become too fast, because longer overlaps recruit more KIF4A, slowing down growth. This emphasizes the self-regulatory nature of the mechanism of antiparallel overlap formation.

*In vitro* self-organized midzone-like microtubule overlaps remain stable for much longer than the few minutes required in cells to complete anaphase and telophase combined [35]. The mechanism discussed here also explains the observed phenotypes of KIF4A depletion in cells [6, 8, 25]. *In vitro* robust antiparallel overlap formation by PRC1 and KIF4A can occur via different pathways: in solution as described here or on surfaces with dynamic or static microtubule pairs [14, 28]. For spindles in cells, yet another pathway variation may be at play, as the metaphase architecture gradually transforms into the anaphase and telophase architecture. It will be interesting to understand to which extent the mechanism proposed here contributes to this transformation in cells.

In conclusion, PRC1 and KIF4A robustly form antiparallel microtubule bundles in solution that recapitulate the architecture of central bundles in the anaphase spindle. It will be interesting to add other central spindle components to expand the functionality of our system, mimicking more closely the central anaphase midzone.

## STAR★Methods

### Key Resources Table

**Table:** 

REAGENT or RESOURCE	SOURCE	IDENTIFIER
**Bacterial and Virus Strains**		

Bacterial strain for molecular cloning: *Escherichia coli* DH5α	EMBL	Strain name: DH5α
Bacterial strain for generating bacmids: *Escherichia coli* DH10MultiBac	Gift from Imre Berger [36]	Strain name: DH10MultiBac

**Chemicals, Peptides, and Recombinant Proteins**		

PRC1-SNAP	This study	Corresponding recombinant DNA: pFF01
PRC1-mGFP	This study	Corresponding recombinant DNA pJG175
KIF4A	This study	Corresponding recombinant DNA: pFF02
KIF4A-mGFP	This study	Corresponding recombinant DNA: pFF03
KIF4A-mBFP	This study	Corresponding recombinant DNA: pFF04
Pig brain tubulin	Purified according to [37]	N/A
Catalase	Sigma-Aldrich	Cat#: C40
Glucose Oxidase	Serva	Cat#: 22778.01
Bovine Serum Albumin	Sigma-Aldrich	Cat#: 05470
Κ-casein	Sigma-Aldrich	Cat#: C0406
Neutravidin	LifeTechnologies	Cat#: A2666
(3-Glycidyloxypropyl)trimethoxy-silane	Sigma-Aldrich	Cat#: 440167
Biotin-CONH-PEG-NH_2_ (3000 Da)	Rapp Polymere Gmbh	Cat#: 133000-25-20
HO-PEG-NH_2_ (3000 Da)	Rapp Polymere Gmbh	Cat#: 103000-20
Poly-L-lysine polyethylene glycol (PLL-PEG)	SUSOS	Cat#: PLL(20)-g[3.5]- PEG(2)

**Experimental Models: Cell Lines**		

Insect cells for recombinant protein expression: *Spodoptera frugiperda* 21 (Sf21)	EMBL	Cell line name: Sf21

**Recombinant DNA**		

pFF01 (pFastBacHTa_humanPRC1-SNAP)	This study	cDNA from BioScience (NCBI Reference Sequence: NM_003981.2)
pJG175 (pFastBacHTa_humanPRC1-mGFP)	This study	cDNA from BioScience (NCBI Reference Sequence: NM_003981.2)
pFF02 (pFastBac1_KIF4A-TEV-10His)	This study	cDNA from Origene (NCBI Reference Sequence: NM_012310.2)
pFF03 (pFastBac1_KIF4A-mGFP)	This study	cDNA from Origene (NCBI Reference Sequence: NM_012310.2)
pFF04 (pFastBac1_KIF4A-mBFP)	This study	cDNA from Origene (NCBI Reference Sequence: NM_012310.2)

**Software and Algorithms**		

FiJi for image analysis	NIH, USA	https://fiji.sc/
Origin for statistical analysis and least-squares fitting	OriginLab, USA	http://www.OriginLab.com
Python for data analysis	CWI, the Netherlands	https://www.python.org/
Cytosim	[30]	https://github.com/nedelec/cytosim

**Other**		

Ni-TED resin	Macherey-Nagel	Cat#: 745200.12
StrepTrap HP column	GE Healthcare	Cat#: 28907547
PD-10 desalting column	GE Healthcare	Cat#: 17-0851-01
Superose 6 10/30 column	GE Healthcare	Cat#: 29-0915-96
Vivaspin concentrator	Sartorius	Cat#: VS15RH21

### Contact For Reagent and Resource Sharing

Further information and requests for resources and reagents should be directed to and will be fulfilled by the Lead Contact Thomas Surrey (thomas.surrey@crick.ac.uk).

### Experimental Model and Subject Details

*Escherichia coli* bacterial strains DH5a and DH10MultiBac were grown in Luria Bertani (LB) medium in the appropriate antibiotics.

For expression of recombinant proteins in insect cells we used Spodoptera frugiperda strain Sf21 grown in suspension Sf-900TM III SFM (1x) Serum Free Medium (GIBCO). Absence of mycoplasma contamination was verified regularly.

### Method Details

#### Molecular cloning

Human PRC1 isoform 1 (NM_003981.2, BioScience) was subcloned into a modified pFastBacHTa vector [14] containing a sequence coding for an N-terminal hexa-histidine-tag separated from the PRC1 sequence by a TEV protease cleavage site, and a C-terminal SNAP-tag (NEB) or monomeric GFP, creating PRC1–SNAP-His and PRC1-mGFP-His expression constructs. PRC1 without a SNAP-tag was cloned similarly, omitting the SNAP sequence.

Full length human KIF4A (NM_012310.2, OriGene) was cloned into a modified pFastBac1, containing a sequence coding for a C-terminal monomeric GFP or BFP (mGFP or mBFP) and a C-terminal deca-histidine-tag separated from the fluorescent protein sequence by a TEV protease cleavage site, thus generating KIF4A-mGFP-His and KIF4A-mBFP-His expression constructs. A non-fluorescent KIF4A was cloned similarly, omitting the fluorescent protein sequence.

#### Protein Purification

Recombinant KIF4A-His, KIF4A-mGFP-His or KIF4A-mBFP-His were expressed in *Sf*21 insect cells. Harvested cells were resuspended in ice-cold KIF4A lysis buffer (50 mM NaPi, 350 mM KCl, 2 mM imidazole, 1 mM MgCl_2_, 1 mM EDTA, 10 mM 2-mercaptoethanol (ME), 0.2 mM ATP, 50 mM glutamate, 50 mM arginine, pH 7.5) supplemented with protease inhibitors (Roche). Resuspended cells were lysed by douncing on ice and the lysate was clarified by ultracentrifugation (183,632 *g,* 30 min, 4°C). Clarified lysate was then incubated with 1.5 g Ni-TED resin (Macherey-Nagel) for 2 h at 4°C on a spinning wheel. This was then loaded into an empty 4 mL gravity column, and the column washed with 80 mL KIF4A lysis buffer, followed by elution in KIF4A elution buffer (50 mM NaPi, 350 mM KCl, 300 mM imidazole, 1 mM MgCl_2_, 1 mM EDTA, 10 mM ME, 0.2 mM ATP, 50 mM glutamate, 50 mM arginine, pH 7.5). The elution buffer was then exchanged for KIF4A gel filtration buffer (50 mM NaPi, 350 mM KCl, 1 mM MgCl_2_, 1 mM EDTA, 2 mM DTT, 0.2 mM ATP, 50 mM glutamate, 50 mM arginine, pH 7.5) using PD-10 columns (GE Lifesciences), and the C-terminal His-tag was removed by overnight TEV protease cleavage on ice. The protein was then passed over the Ni-TED column again to remove any uncleaved protein and the flow through was concentrated (up to 5 mg/mL) using a Vivaspin concentrator (Sartorius; VS15RH21). The concentrated solution was gel filtered using a Superose 6 10/30 column (GE Healthcare) equilibrated with gel filtration buffer. Peak protein fractions were pooled, and concentrated to a concentration of 1.25 mg/mL, as measured using a NanoDrop ND-1000 Spectrophotometer. Glycerol was added to a final concentration of 20% (v/v), and the solution was ultracentrifuged (278,088 g, 15 min, 4°C), aliquoted and stored in liquid nitrogen. Yields were ∼1.5 mg of purified protein from a 600 mL insect cell culture. Purity of proteins was verified by Coomassie-stained SDS gel electrophoresis (Figure S6).

PRC1, PRC1-SNAP and PRC1-mGFP were expressed in *Sf21* cells and purified as KIF4A, but with the following corresponding buffers: PRC1 lysis buffer (50 mM NaPi, 500 mM KCl, 2 mM imidazole, 3 mM EDTA, 10 mM ME, pH 7.5); PRC1 elution buffer (50 mM NaPi, 500 mM KCl, 400 mM imidazole, 3 mM EDTA, 10 mM ME, pH 7.5); PRC1 gel filtration buffer (50 mM NaPi, 500 mM KCl, 3 mM EDTA, 2 mM DTT, pH 7.5). SNAP labeling was performed overnight in parallel with the TEV cleavage by adding SNAP-Surface Alexa Fluor-546 (NEB) dissolved in DMSO, so as to have a 2:1 ratio of label:PRC1-SNAP. The labeling ratio was 0.91 and yields were ∼1.5 mg of protein from a 600 mL insect cell culture, as assessed using a NanoDrop ND-1000 Spectrophotometer. Purity of proteins was verified by Coomassie-stained SDS gel electrophoresis (Figure S6).

Porcine brain tubulin was purified as described [37]. Purified tubulin was recycled and labeled with Alexa647-*N*-hydroxysuccinimide ester (NHS; Sigma-Aldrich), or biotin-NHS (Thermo Scientific), as described previously [38].

#### Preparation of stabilized microtubules

Short biotinylated GMPCPP-stabilized microtubules were polymerized from a mixture of Alexa647-tubulin (12.1 μM, labeling ratio 0.1) and biotin-tubulin (6 μM) in the presence of 0.5 μM GMPCPP (Jena Bioscience) in 60 μl of BRB80 (80 mM PIPES, 1 mM MgCl_2_, 1 mM EGTA) for 1 h at 37°C, centrifuged at 17,000 x *g* for 15 min, washed with warm BRB80 (37°C), centrifuged again at 17,000 x *g* for 10 min, resuspended in 50 μl BRB80 and kept at room temperature.

Long biotinylated GMPCPP-stabilized microtubules were polymerized from a mixture of Alexa647-tubulin (1.7 μM, labeling ratio 0.1) and biotin-tubulin (0.5 μM) in the presence of 0.3 μM GMPCPP (Jena Bioscience) in 300 μl of BRB80 for 2 h at 37°C, centrifuged at 17,000 x *g* for 15 min, washed with warm BRB80 (37°C), centrifuged again at 17,000 x *g* for 10 min, resuspended in 30 μl BRB80 and kept at room temperature.

#### Binding to immobilised stabilized microtubules

Biotin-polyethylene glycol (biotin-PEG)-passivated coverslips were produced as described previously [39, 40], using 10% biotin-PEG-NH_2_ and 90% HO-PEG-NH_2_ (both 3000 Da; Rapp Polymere). 50 μL 5% (m/v) Pluronic F-127 (Sigma) was flowed through a flow chamber composed of a biotin-PEG-passivated coverslip and a poly-L-lysine-polyethylene glycol (PLL-PEG)-passivated glass slide separated by two double sticky tapes [39] and left for 10 min at room temperature. 2 × 50 μL assay buffer (AB; 80 mM PIPES, 85 mM KOAc, 4.5 mM MgCl_2_, 1 mM EGTA, 0.005% Brij-35, 10 mM ME, 33 mM glucose, 0.15% (m/v) methyl cellulose, pH 6.8) supplemented with 0.2 mg/mL κ-casein (κ-AB) was flowed through the flow chamber at room temperature, and the flow chamber was then transferred onto an ice-cold metal block, and 50 μL of 50 μg/mL NeutrAvidin (LifeTechnologies) dissolved in κ-AB was flowed in, followed by 3 min incubation. The flow chamber was then taken off the ice, and 2 × 50 μL AB was flowed through before incubating for 3 min with an appropriate dilution of stabilized GMPCPP microtubules. After seed incubation, 2 × 50 μL AB was flowed through the flow chamber at room temperature, followed by 50 μL of the final reaction mix: AB supplemented with 1 mM GTP, 2 mM ATP, 1 mg/mL glucose oxidase, 0.5 mg/mL catalase, and with 1% (v/v) PRC1-Alexa546 and 2.36% (v/v) KIF4A-mBFP added at the appropriate concentrations in their respective storage buffers (see above) to yield final protein concentrations as stated in the Figure Legends. For controls without PRC1 or KIF4A, only the respective storage buffer was added. The flow chamber was immediately sealed with vacuum grease (Beckman Coulter) and placed on the TIRF microscope in a temperature-controlled box kept at 30°C.

Control experiments at lower and higher ionic strength were performed as above, but AB was replaced by low ionic strength buffer (LB; 80 mM PIPES, 5% (m/v) sucrose, 4.5 mM MgCl_2_, 1 mM EGTA, 71.5 mM ME, 33 mM glucose, 0.15% (m/v) methyl cellulose, pH 6.8) or high ionic strength buffer (HB; 80 mM PIPES, 85 mM KCl, 85 mM KOAc, 4.5 mM MgCl_2_, 1 mM EGTA, 0.005% Brij-35, 10 mM ME, 33 mM glucose, 0.15% (m/v) methyl cellulose, pH 6.8) respectively. κ-AB was replaced by the corresponding κ-LB and κ-HB.

#### Self-organization of minimal anaphase midzones

Flow chambers were prepared as described above. 10 min after flowing 50 μL 5% (m/v) Pluronic F-127 (Sigma) through the flow chamber at room temperature, 3 × 50 μL κ-AB was flowed through, and the flow chamber was transferred onto ice for 3 min. Then 2 × 50 μL AB was flowed through the chamber at room temperature, followed by 50 μL of the final reaction mix: AB supplemented with 1 mM GTP, 2 mM ATP, 1 mg/mL glucose oxidase, 0.5 mg/mL catalase, 12.5 μM Alexa647-tubulin (labeling ratio: 0.1), and with 1% (v/v) PRC1-Alexa546 and 2.36% (v/v) KIF4A-mBFP added in their respective storage buffers at the appropriate concentrations to yield final protein concentrations as stated in the Figure Legends. For controls without PRC1 or KIF4A, only storage buffer was added. For data shown in Figures 1C, 1D, 3A, and 3B, final solutions were ultracentrifuged (278,088 g, 15 min, 4°C) before flowing into the chamber. The flow chamber was immediately sealed with vacuum grease (Beckman Coulter) and placed on the TIRF microscope in a temperature-controlled box kept at 30°C. Self-organizing antiparallel microtubule bundles were free to diffuse, but were kept close to the glass surface by the low concentrations of the crowding agent methyl cellulose, allowing convenient TIRF microscopy and confocal microscopy imaging.

#### Total internal reflection fluorescence (TIRF) microscopy

TIRF microscopy experiments were performed on a TIRF microscope based on a Nikon Ti-E frame with a 100x 1.49 N.A. Nikon objective lens and 360° TIRF illumination (Cairn Research, Faversham, UK). For the recording of triple-color images, Alexa647-tubulin (640 nm excitation) and KIF4A-mBFP (405 nm excitation) were recorded simultaneously in separate channels, PRC1-Alexa546 (561 nm excitation) was recorded separately, using Andor iXon Ultra 888 EMCCD cameras (exposure time 100 ms).

For imaging binding to single immobilised microtubules, triple-color images were captured ∼12 min after adding the final mixture to the flow chamber.

To record triple-color time-lapse videos of self-organization assays, imaging starting ∼2 min after adding the protein mixture to the flow chamber and lasted for 37.5 min. The time interval between images was 4.5 s for data in Figures 2 and S2, and 18 s for all other time course data. One time-course per sample was recorded.

After recording time courses of minimal midzone self-organization, several additional images per sample were recorded outside the area used for imaging the time course. These data were used to investigate the dependence of the end state of self-organization on the KIF4A and PRC1 concentrations (Figures 4, 5C, 5D, and S6).

Imaging of single dynamic microtubules started ∼2 min after adding the protein mixture to the flow chamber. Experiments were recorded over 12.5 min, with a time interval of 1.5 s per frame.

#### Photobleaching assays and confocal microscopy

To image bleach marks in microtubule segments outside of antiparallel overlaps of minimal midzones, samples were prepared as described above for the self-organization of minimal midzones. The final solution contained 12.5 μM Alexa647-tubulin, 5 nM unlabelled PRC1 and 50 nM unlabelled KIF4A. Experiments were performed on a spinning disk confocal microscope comprising a Yokogawa CSU M1 spinning disk on a Zeiss Axio Observer Z1 with automated FRAP unit (3i, London, UK). After introducing the final solution, flow chambers were sealed and incubated on the microscope for 15 min at 30°C. Time-lapse videos were then recorded over 37.5 min, with 4.5 s time intervals between images, using a 100x oil objective. Imaging started ∼10 min after adding the protein mixture to the flow chamber and bleach marks were made on microtubules outside the central overlap using the 640 nm laser ∼10 min after the start of imaging.

For fluorescence recovery after photobleaching (FRAP) experiments, self-organization experiments were performed with final solutions that contained 12.5 μM unlabelled tubulin and 20 nM PRC1 and 50 nM KIF4A, one of which was labeled with mGFP, the other one unlabelled. Entire overlaps of reconstituted minimal midzone bundles with either KIF4A-mGFP or PRC1-mGFP present were bleached using the 488 nm laser after 40 min, and experiments were recorded with 5 s time intervals between images, over a period of 10-15 min post-bleaching. The total fluorescence intensity of the bleached area was measured and plotted over time to assay for fluorescence recovery of the KIF4A-mGFP or PRC1-mGFP fluorescence.

### Quantification and Statistical Analysis

#### I. Image analysis

##### Estimating polymerized microtubule mass and bundle numbers

Microtubule bundles growing in the presence of 12.5 μM Alexa647-tubulin and 5, 10, 20 or 50 nM PRC1-Alexa546 were analyzed. For each video, the number of individual bundles within the field of view was manually counted at 230 s, before considerable bundle fusion occurred. In addition, the total integrated tubulin intensity above a manually set threshold was measured at 675 s.

##### Bundle tracking and kymograph generation

Image analysis was performed using custom macros written in Fiji/ImageJ (https://fiji.sc/). In time-lapse videos of minimal anaphase midzone formation, the microtubule background fluorescence was subtracted using a 250 pixels rolling ball process. The PRC1 channel was used to track the center of each bundle over time: the PRC1 channel was duplicated, a 1 pixel Gaussian blur applied, and the user manually selected the intensity threshold to roughly define the overlap regions. The ‘Analyze Particles’ plugin was run to further refine the position of all overlap regions in each frame. The ‘DropletTracker2′ plugin (https://github.com/ottobonn/DropletTracker) was used to link corresponding overlap regions between each frame. Overlaps were rejected if tracked for less than 50 or 200 image frames for videos recorded with 18 s or 4.5 s time intervals between image frames, respectively, or if the overlaps were within 130 pixels of the edge of the image. For each remaining overlap, the central coordinates and orientation were used to obtain an intensity profile along the length of the bundle, averaged over a width of 25 pixels, in each frame for each fluorescence channel. A composite kymograph was generated from these intensity profiles.

##### Analysis of kymographs

For each kymograph generated, the size of the PRC1-labeled antiparallel microtubule overlap and the intensity in each fluorescence channel within the overlap was extracted: For each line of the kymograph (corresponding to an overlap profile in a single frame) a boxcar sub-region of 6 adjacent lines was created. Within this sub-region the Moments auto-threshold method (https://imagej.net/Auto_Threshold#Moments) was applied to the PRC1 channel to identify the overlap. The overlap length for the sub-region was calculated as the total area above threshold divided by 6. The total intensities of pixels above this threshold were measured for all three channels, to give total integrated tubulin, PRC1, and KIF4A intensities. For the analysis of images in time lapse videos, the sub-region was moved down the kymograph by one line (frame), the overlap and intensities recalculated, and the process repeated for all frames.

Time course data from 17 overlaps produced in the presence of 20 nM PRC1-Alexa546, 50 nM KIF4A-mBFP and 12.5 μM Alexa647-tubulin were used to calculate the average time course of overlap length and total protein intensities in the overlap region.

Peak overlap lengths and their corresponding properties were determined from a 90 s moving average of the raw time lapse data for each overlap.

For the statistical analysis of final antiparallel microtubule overlap properties, overlap lengths and total fluorescence intensities were extracted from images taken ∼40 min after initiation of microtubule nucleation for more than 93 overlaps per condition. The investigated conditions were 12.5 μM Alexa647-tubulin and the following ten combinations of PRC1-Alexa546 / KIF4A-mBFP concentrations (in nM/nM): 5/ 5, 5/10, 5/50, 10/5, 10/10, 10/50, 20/5, 20/10, 20/50, 50/50. From these data, the statistical properties of overlap length and total protein intensity in the overlap were calculated for each condition. Pearson correlation coefficients were calculated for each pair of overlap properties per condition. Mean correlation coefficients were then calculated averaging over all conditions.

The absolute number of microtubules in the end overlap was calculated using the total tubulin intensity in the overlap divided by the overlap length. The scaling factor to convert the resulting average tubulin intensity per length to microtubule number was estimated from kymographs of bundles with few microtubules, where individual microtubules could be identified.

##### Determination of microtubule growth speeds

Microtubule growth speeds were determined for different assay conditions using kymographs generated from tracked bundles as described above. The extensions of individual microtubules were identified on the kymographs and traced over periods of approximately constant growth. For the determination of plus- and minus-end speeds in the KIF4A control, only initial periods of growth from the seed were chosen, where there was no evidence of KIF4A binding. Speeds were calculated using a custom macro in ImageJ.

#### II. Computer Simulations

We simulated antiparallel microtubule crosslinking by PRC1 and sliding by KIF4A using Cytosim, an Open Source simulation engine based on Brownian dynamics [30]. For parameter sets, see Table S1. Simulation files are in Data S1.

##### Modeling of PRC1

A PRC1 dimer is simulated as two individual heads connected by an elastic linker, that can attach to different microtubules (Figure S5A). PRC1 dimers do not associate with each other. When they are unattached, PRC1 molecules diffuse in solution, and each head can bind to the microtubule with a constant rate *k*_*on*_ if the distance between the molecule and the microtubule is smaller than a given binding distance *d*_*b*_. When only one of the heads is attached, the other head can attach to a different microtubule as it would from solution (Figure S5A, 1). When a PRC1 molecule is crosslinking two microtubules, a displacement from the resting length of the spring produces a force *f*_*c*_ that is transmitted to the microtubules. This force increases the unbinding rate exponentially (Figure S5A, 4):

(Eq. 1) $$k_{\text{off}}=k_{\text{off}}^{0}\;\text{exp}\left(\|\vec{f_{c}}\|/f_{u}\right)$$

where the unbinding force *f*_*u*_ is a parameter. The projection of this force in the direction of the microtubule influences the movement of the heads along the microtubule. We modeled these diffusible crosslinkers in Cytosim on a lattice of unit space *a* = 8 nm, which is the tubulin heterodimer length. As observed, we assume that PRC1 can diffuse on this lattice by hopping to adjacent sites with a rate *k*_*0*_ = *D* / *a*^*2*^, where *D* is the diffusion constant of the crosslinkers on a microtubule. Moreover, a crosslinker cannot move to a position which is already occupied. In the simulation, stepping out of the microtubule at the plus- or minus end is not allowed (Figure S5A, 5), since accumulation at the ends has been observed experimentally [31]. In the model, the fall-off probability of PRC1 from any position is the same for simplicity. In the absence of an external force, crosslinkers would hop with equal probability in both directions, resulting in an unbiased diffusion process. However, the difference of energy of the different states affects the upstream and downstream rates such that *h*_*+*_ ≠ *h*_*-*_ (Figure S5A, 6). Thermodynamic considerations dictate that for any pair of states (*a*,*b*) corresponding to different potential energy *U*_*a*_ and *U*_*b*_, transition rates should satisfy Arrhenius law:

(Eq. 2) $$\frac{h_{a\to b}}{h_{b\to a}}=\text{exp}\left(\epsilon \right)\;\text{with}\;\epsilon =\left(U_{a}-U_{b}\right)/k_{B}T$$

The solution that was used to model this process, from [41] is:

(Eq. 3) $$h_{a\to b}=\;\frac{\varepsilon }{1-e^{-\varepsilon }}k_{0}\;\text{and}\;h_{b\to a}=\;\frac{\varepsilon }{e^{\varepsilon }-1}k_{0}$$

From the current position of a linker, we calculate the plus and minus end directed rates *h*_+_ and *h*_−_ from the Δ*U* corresponding to the differences in elastic energy in the linker between the future state and the current state, and applying Eq. 3. In the continuous limit of small lattice unit (*a*), we define *d* = *D* / *k*_*B*_*T* as a mobility coefficient for bound PRC1 molecules; the average speed of a diffusive head under a given force (corresponding to Δ*U* = −*af*_*c*_) reads:

(Eq. 4) $$v_{c}\left(f_{c}\right)=a\left(h_{+}-h_{-}\right)=d\;f_{c}$$

##### Modeling of KIF4A

Motors are composed of one head, that can bind and unbind from microtubules as described above (Figure S5A, 1). KIF4A also binds to discrete sites regularly spaced every 8 nm on microtubules, which are distinct from the PRC1 binding sites. This assumption agrees with experimental observations showing that kinesin-4 can move on microtubules where the PRC1 density was so high that individual PRC1 molecules did not diffuse [14]. In our model, we tried two assumptions for KIF4A: either it is not allowed to fall off at microtubule plus-ends, or it falls of immediately (parameter set 3). KIF4A can associate with PRC1 from the microtubule or from solution (Figure S5A, 2). This interaction is represented by an elastic link between the KIF4A head, and the center of the PRC1 linker, as described below. When it is not bound to PRC1, KIF4A moves on the lattice toward the plus end with a rate *v*_*0*_ / *a*, given by its maximum speed *v*_*0*_ and the lattice spacing *a*. When it is bound to PRC1, the stochastic plus end stepping rate is affected by the antagonistic force in the linker (*f*_*m*_ > 0) (Figure S5A, 7), and follows the linear force-velocity relation that was measured for kinesin [42], where *f*_*s*_ is the stall force of the motor:

(Eq. 5) $$h_{+}=\frac{v_{0}}{a}\left(1-\frac{f_{m}}{f_{s}}\right)$$

##### Modeling of the interactions

KIF4A can attach to PRC1 from the microtubule, when a PRC1 head and a KIF4A head are on adjacent sites on the lattice, with a rate *k*_*am*_ (Figure S5A, 2). KIF4A can also attach from solution to PRC1 on the microtubule or to PRC1 in solution following second-order reaction kinetics with rate *k*_*as*_ (Figure S5A, 2). KIF4A that is bound to PRC1 can bind to microtubules as it does from solution. The interaction is represented by an elastic link between the KIF4A head, and the center of the PRC1 linker. The three heads are then connected to a dragless junction by elastic linkers of equal stiffness, and the position of the junction at every time point is given by the force balance of the connected links. To calculate the force applied to each head of the complex when all three are attached, we use the following equivalence: Let *P*_1_, *P*_2_ and *P*_3_ be the positions in space of the two heads of the PRC1 molecule and the KIF4A head, and *X* the position of the dragless junction. If the stiffness of all linkers is the same (*k*), the force balance reads:

(Eq. 6) $$0=k\left(P_{1}-X\right)+k\left(P_{2}-X\right)+k\left(P_{3}-X\right)$$

(Eq. 7) $$X\;=\;\frac{1}{3}\left(P_{1}+P_{2}+P_{3}\right)$$

Since *P*_1_ is only linked to *X*, the force on *P*_1_ is *k* (*X* - *P*_1_). If we substitute *X*, we get:

(Eq. 8) $$f_{1}\;=\;\frac{k}{3}\left[\left(P_{2}-P_{1}\right)+\left(P_{3}-P_{1}\right)\right]$$

And similarly, for *P*_2_ and *P*_3_. Therefore, the situation is comparable to having the three heads linked to each other by springs of elastic constant *k*/3 (Figure S5B). In case only two heads of the complex are bound, the effective stiffness acting between the two heads is *k*/2 (Figure S5B). The force in the link between KIF4A and PRC1 affects the detachment rate of KIF4A from PRC1 and the unbinding of KIF4A from the microtubule with forces characteristic of the interaction between KIF4A and PRC1 (*f*_*d*_), and KIF4A and the microtubule (*f*_*u*_) as in Equation 1. In the simulation we do not include the scenario in which a complex of PRC1-KIF4A is only attached to the microtubule through the KIF4A head, because experimentally KIF4A binding is strongly enhanced by the presence of PRC1; therefore, when the complex PRC1-KIF4A binds from solution, it only does so by first attaching a PRC1 head. When in a complex in which one PRC1 head is bound to the microtubule there is unbinding of that head from the microtubule, the KIF4A detaches from PRC1.

##### Running the simulations

Initially the two microtubules overlap completely. First, PRC1 alone is simulated for 10 min to reach binding equilibrium. KIF4A is then added, and the simulation is continued, recording the overlap length, the number of crosslinking PRC1 and associated KIF4A molecules for 5 min.

#### III. Theory

##### Microtubule sliding mechanism

The PRC1-KIF4A system can produce effective microtubule sliding. Here we derive the steady-state speed of sliding caused by *N* complexes of KIF4A-PRC1 in which all heads are attached, and crosslink two antiparallel microtubules. Since the system is symmetrical, we can focus only on one of the microtubules. If we name *m* the motor, *a* the PRC1 head that is attached to the same microtubule as the motor, and *b* the PRC1 head attached to the opposite microtubule (Figure S5C); using the equivalence in Figure S5B to derive the force, we find these relationships for the speeds on the microtubule lattice:

(Eq. 9) $$v_{m}=v_{0}\left(1-\frac{f_{ma}+f_{mb}}{f_{s}}\right)$$

(Eq. 10) $$v_{a}=d\;\left(\;f_{ma}-f_{ab}\;\right)$$

(Eq. 11) $$v_{b}=d\;\left(\;f_{mb}+f_{ab}\;\right)$$

where *f*_*ij*_ represents the projection of the force between *i* and *j* on the microtubule.

The speed of a microtubule with respect to the other, *v*_*T*_, is given by:

(Eq. 12) $$v_{T}=\frac{2N}{\gamma }\;\left(\;f_{mb}+f_{ab}\;\right)$$

where γ is the drag of the fluid against the microtubule, and *N* the number of PRC1-KIF4A

complexes. In the steady state *a* and *m* move at the same speed, and the speed of *b* is given

by the sum of the movement of *b* on the lattice plus the displacement of the microtubules with respect to each other, therefore:

(Eq. 13) $$v_{m}=v_{a}=v_{b}+v_{T}$$

We also have by definition of the forces:

(Eq. 14) $$f_{mb}=\;f_{ma}+\;f_{ab}$$

For the known parameters of the system, we estimate that $v_{0}/f_{s}\ll \;d$, and given this simplification, we obtain the following expression for the speed of sliding:

(Eq. 15) $$\frac{v_{0}}{v_{T}}=1+d\;\frac{\gamma }{2N}$$

The sliding speed is thus proportional to the unloaded speed of KIF4A, and depends on the number of PRC1- KIF4A complexes. With high amounts of complexes, the speed is simply $v_{T}=v_{0}$. In the other limit where *2N < dγ*, the speed is proportional to N: $v_{T}=v_{0}\frac{2N}{d\gamma }$.

The product dγ is the ratio between the drag of the microtubules against the fluid (γ) and the drag of the diffusive tails of PRC1 on the microtubule (1/d), effectively represents how the force generated by the motor is used: it is the ratio between the fraction of tension used for microtubule sliding, versus what is released by diffusion of PRC1 heads.

### Data and Software Availability

The computational model was implemented in Cytosim, publicly available at https://github.com/nedelec/cytosim. The Cytosim version specific to this study with additional code and source data (simulation configuration files and documentation) relating to Figures 6 and S5 is provided (Data S1).

## Supplemental Information

Document S1. Figures S1–S6 and Table S1

Data S1. Simulation Files, Related to STAR Methods This ZIP contains the source code of Cytosim used for this study, and all the configuration files needed to reproduce the simulation figures. Please, refer to the README file included in the package.

Document S2. Article plus Supplemental Information
