# Supplementary material for: Self-Organization of Minimal Anaphase Spindle Midzone Bundles
Source: Curr Biol. 2019 Jul 8;29(13):2120–2130.e7. doi: 10.1016/j.cub.2019.05.049 (PMC6616649; doi:10.1016/j.cub.2019.05.049)
Supplement: Document S1. Figures S1–S6 and Table S1 [file mmc1.pdf]

Current Biology, Volume *29*

Supplemental Information

Self-Organization of Minimal Anaphase Spindle Midzone Bundles

Jonathon Hannabuss, Manuel Lera-Ramirez, Nicholas I. Cade, Franck J. Fourniol, François Nédélec, and Thomas Surrey

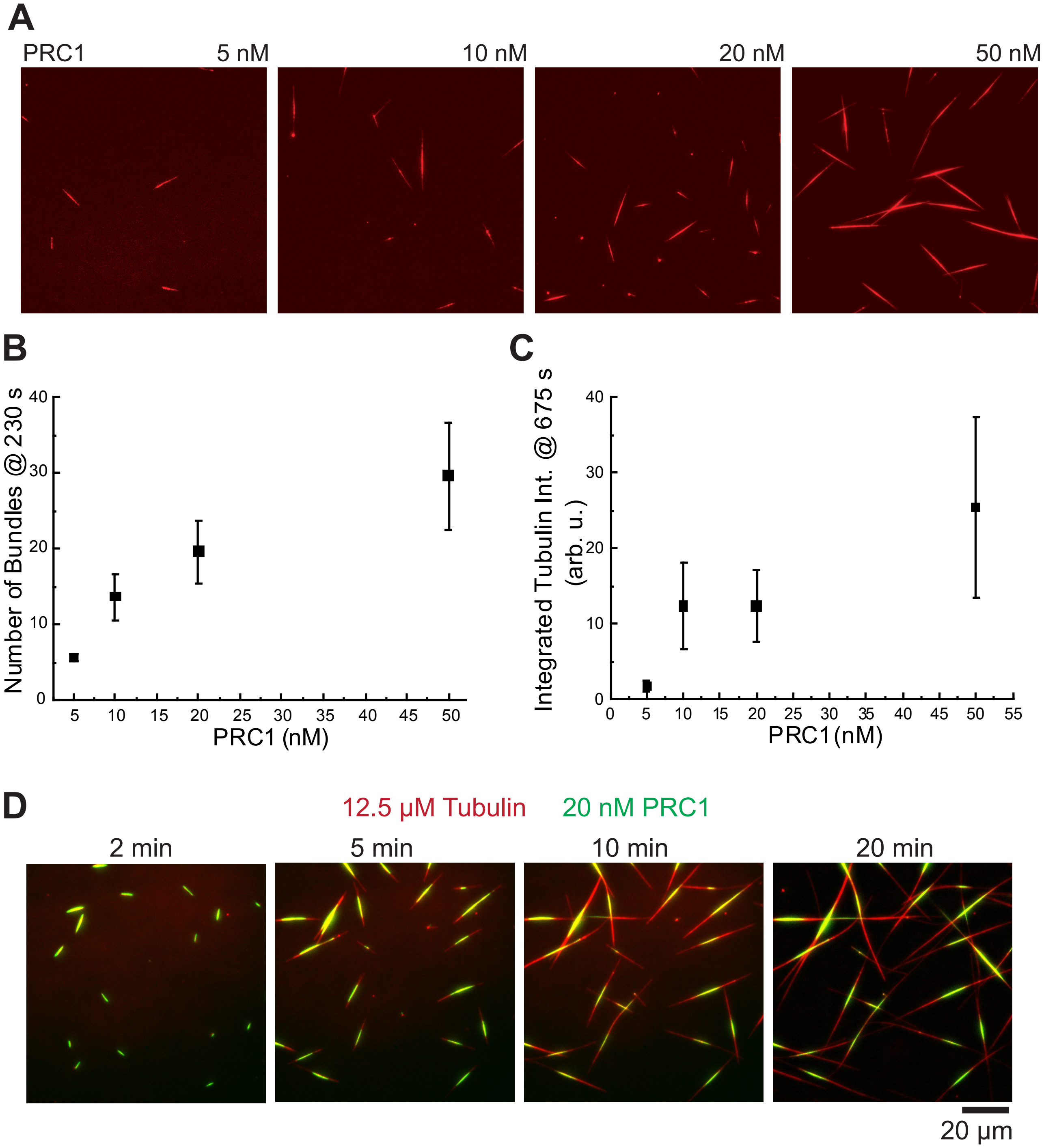
A
PRC1
5 nM
10 nM
20 nM
50 nM
B
Number of Bundles @ 230 s
PRC1 (nM)
C
Integrated Tubulin Int. @ 675 s (arb. u.)
PRC1 (nM)
D
12.5 μM Tubulin
20 nM PRC1
2 min
5 min
10 min
20 min
20 μm

Figure S1. PRC1 bundles, related to Figure 1.

(A) - (C) PRC1 promotes microtubule generation. **(A)** Representative images of the tubulin channel showing individual microtubule bundles, 4 min after initiating microtubule nucleation (before fusion of larger bundles) in the presence of 5-50 nM PRC1-Alexa546 and 12.5 μM Alexa647-tubulin (no KIF4A). **(B)** Number of individual microtubule bundles found in (A) and **(C)** total fluorescence intensity of all imaged bundles 11 min after initiation of nucleation, plotted as a function of the PRC1 concentration. Each point represents the mean value from three movies. Error bars represent standard deviation. **(D)** Kinetics of bundle formation: TIRF microscopy images showing the time course of self-organization of microtubule bundles in the presence of 20 nM PRC1-Alexa546 (green) and 12.5 μM Alexa647-tubulin (red); same condition as in Figure 1F. Times in minutes after initiating microtubule nucleation by a temperature shift to 30°C.

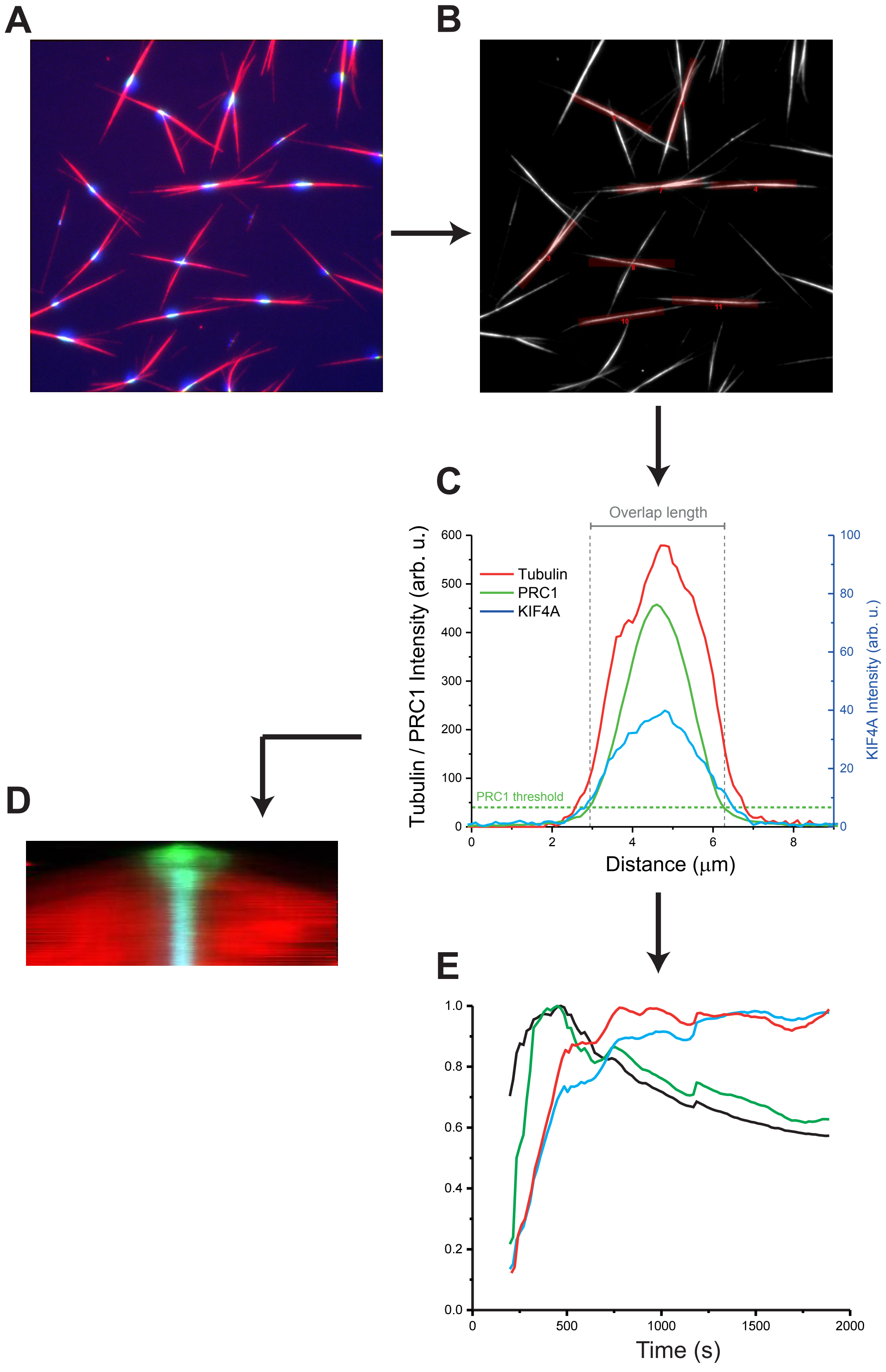
A
B
C
Overlap length
Tubulin / PRC1 Intensity (arb. u.)
KIF4A Intensity (arb. u.)
Tubulin
PRC1
KIF4A
PRC1 threshold
600
500
400
300
200
100
0
100
80
60
40
20
0
0
2
4
6
8
Distance (µm)
D
E
1.0
0.8
0.6
0.4
0.2
0.0
0
500
1000
1500
2000
Time (s)

Figure S2. Quantification of antiparallel microtubule overlap properties, related to Figure 3.

Illustration of the different steps of antiparallel overlap analysis. (**A**) In a time-lapse triple-colour TIRF microscopy movie, antiparallel microtubule bundles are automatically detected, (**B**) their PRC1-rich overlaps are then tracked automatically, (**C**) one-dimensional fluorescence intensity profiles are generated along the entire bundle axis in each image. From these profiles, (**D**) kymographs can be created or (**E**) the time course of the overlap length or of total fluorescence intensities of all three proteins in the overlap region can be calculated. For details see Methods.

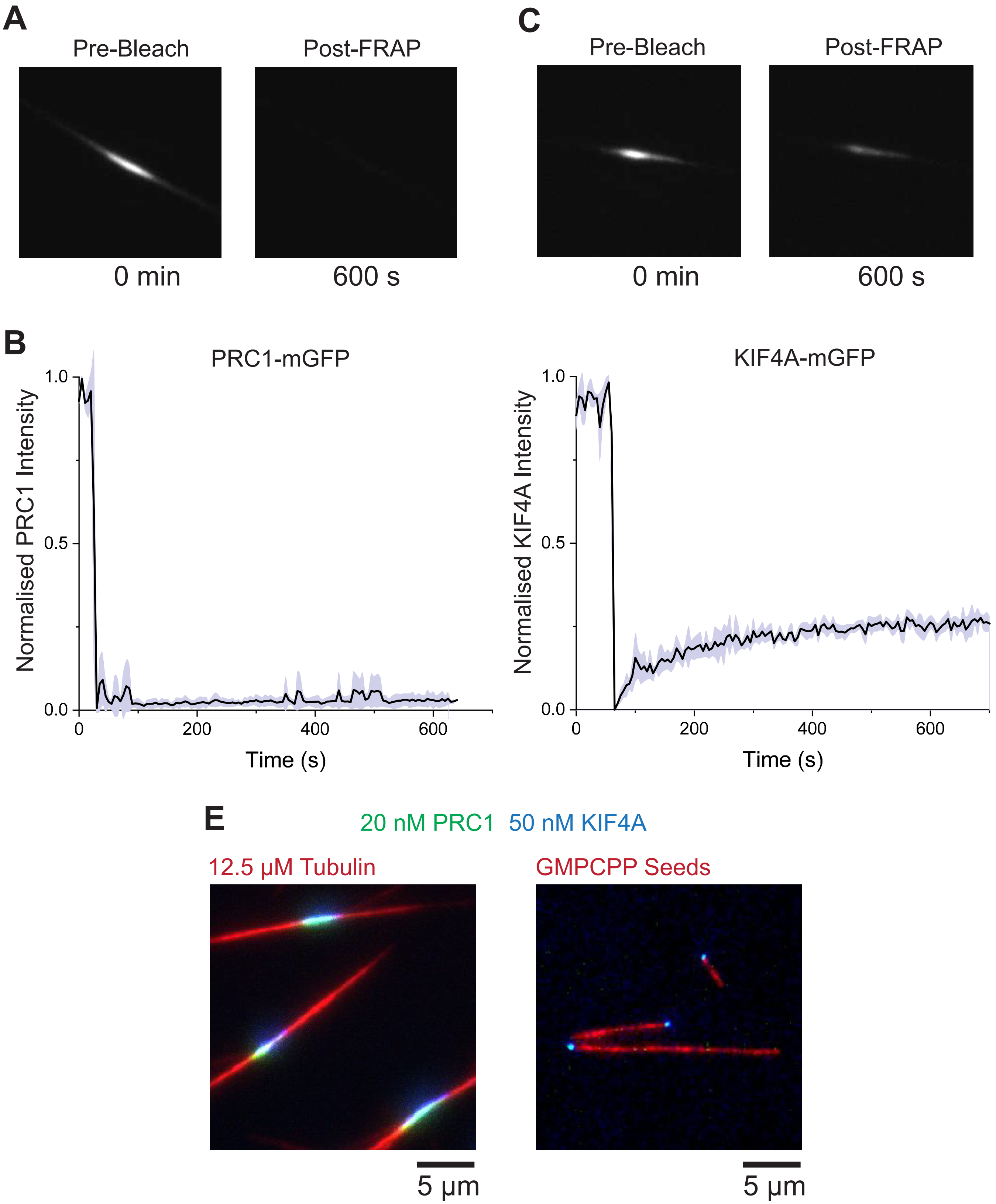

A
Pre-Bleach
Post-FRAP
0 min
600 s
C
Pre-Bleach
Post-FRAP
0 min
600 s
B
PRC1-mGFP
Normalised PRC1 Intensity
1.0
0.5
0.0
0
200
400
600
Time (s)
KIF4A-mGFP
Normalised KIF4A Intensity
1.0
0.5
0.0
0
200
400
600
Time (s)
E
20 nM PRC1
50 nM KIF4A
12.5 μM Tubulin
GMPCPP Seeds
5 μm
5 μm

Figure S3. Minimal midzone bundle characteristics, related to Figures 3 and 4.

(A) - (D) Photobleaching analysis of PRC1 and KIF4A in antiparallel overlaps of minimal midzone bundles. Confocal fluorescence microscopy image of PRC1-mGFP in a minimal midzone bundle before and ~10 minutes after photobleaching the entire bundle using the 488 nm laser. Protein concentrations were 20 nM PRC1-mGFP, 50 nM unlabelled KIF4A and 12.5 µM unlabelled tubulin. **(B)** Time course of the mean PRC1-mGFP fluorescence intensity before and after photobleaching (n = 6); shaded area represents standard deviation. **(C)** Confocal fluorescence microscopy image of KIF4A-mGFP in a minimal midzone bundle before and ~10 minutes after photobleaching the entire bundle. Protein concentrations were 20 nM unlabelled PRC1, 50 nM KIF4A-mGFP, and 12.5 µM unlabelled tubulin. **(D)** Time course of the mean KIF4A-mGFP fluorescence intensity before and after photobleaching (n = 6); shaded area represents standard deviation. For both proteins, photobleaching occurred ~40 minutes after initiating microtubule nucleation by a temperature shift to 30˚C. **(E)** Comparison of overlap and end tag lengths: Representative images showing the overlap regions in microtubule bundles (left), and end tag regions (right) in the presence of 20 nM PRC1-Alexa546, 50 nM KIF4A-mBFP, and either 12.5 µM Alexa647-tubulin (left) or GMPCPP-stabilized seeds (right). Images were taken ~30 minutes after initiating nucleation.

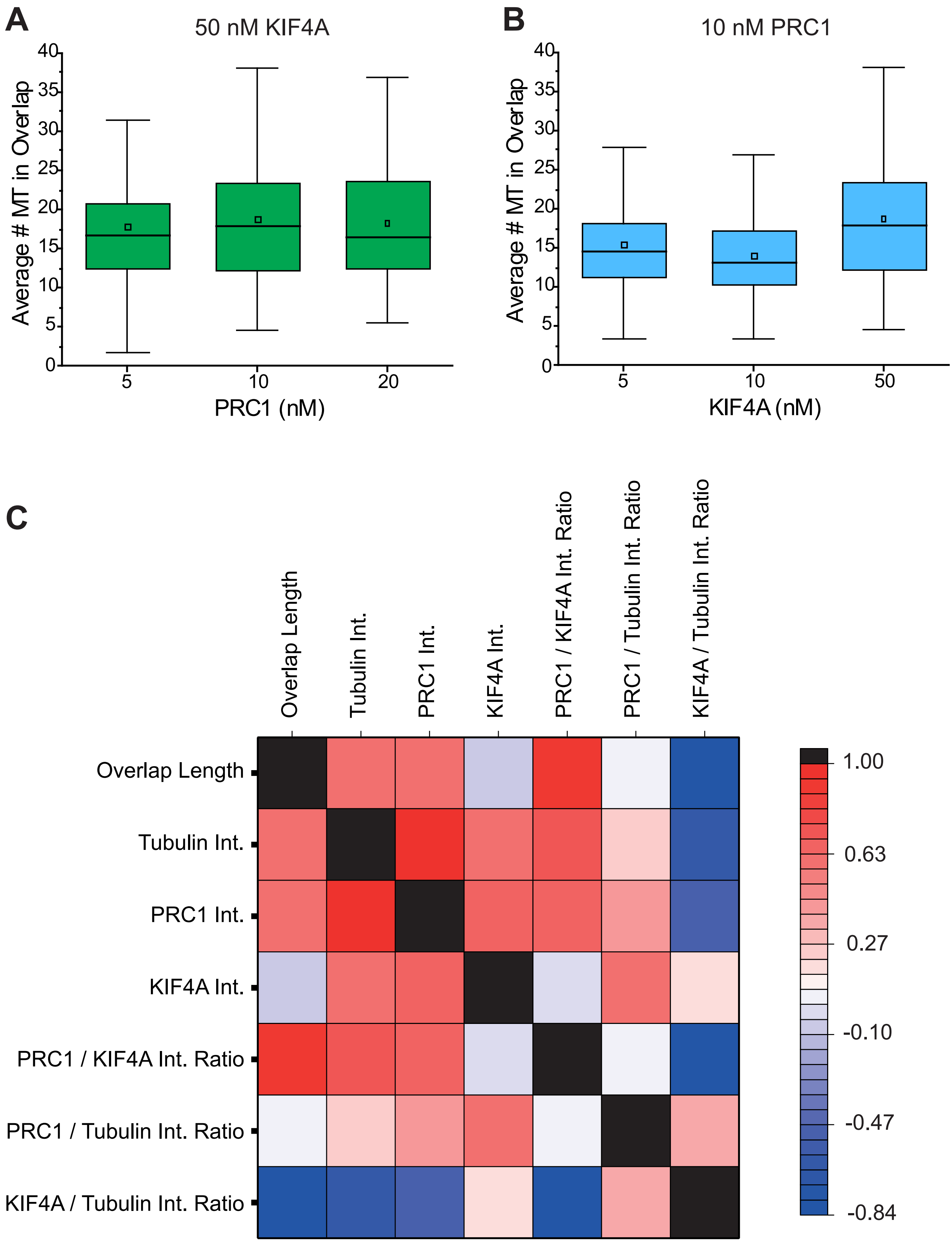
A
50 nM KIF4A
Average # MT in Overlap
0
5
10
15
20
25
30
35
40
5
10
20
PRC1 (nM)
B
10 nM PRC1
Average # MT in Overlap
0
5
10
15
20
25
30
35
40
5
10
50
KIF4A (nM)
C
Overlap Length
Tubulin Int.
PRC1 Int.
KIF4A Int.
PRC1 / KIF4A Int. Ratio
PRC1 / Tubulin Int. Ratio
KIF4A / Tubulin Int. Ratio
1.00
0.63
0.27
-0.10
-0.47
-0.84

Figure S4. Parameter dependencies of microtubule number and overlap length in minimal antiparallel midzone bundles, related to Figures 4 and 5.

(A) Box plots showing the estimated average number of microtubules in final antiparallel microtubule overlaps in the presence of 50 nM KIF4A and varying concentrations of PRC1 (conditions as in Figure 4B). Box represents IQR, whiskers represent range (-outliers), box line = median, square = mean. **(B)** as (A) for 10 nM PRC1 and varying concentrations of KIF4A (conditions as in Figure 4D). **(C)** Correlation matrix of antiparallel overlap properties, presented as a heat map visualising mean Pearson correlation coefficients for all pairs of antiparallel overlap properties measured in minimal midzone bundles ~40 minutes after microtubule nucleation. Mean correlation values were calculated from the same set of experiments exploring a range of PRC1 and KIF4A concentrations used to produce Figure 4C & D, Figure 5A & B. Correlations represent correlations between the mean values (see Figures 4F and 5A/B). Red represents a positive correlation, white a neutral correlation and blue a negative correlation.

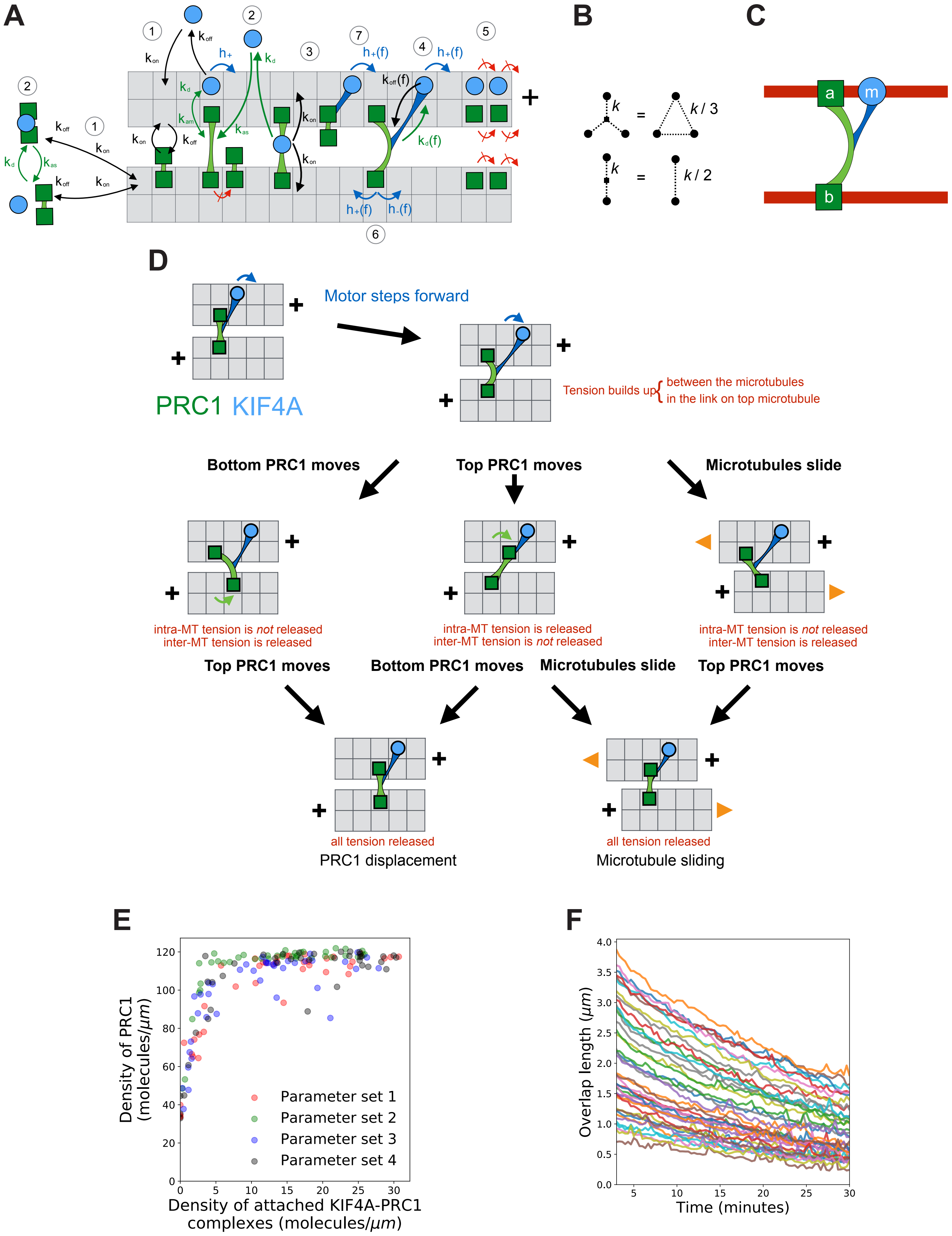

A
B
C
D
Motor steps forward
PRC1 KIF4A
Tension builds up between the microtubules in the link on top microtubule
Bottom PRC1 moves
Top PRC1 moves
Microtubules slide
intra-MT tension is not released
inter-MT tension is released
intra-MT tension is released
inter-MT tension is not released
intra-MT tension is not released
inter-MT tension is released
Top PRC1 moves
Bottom PRC1 moves
Microtubules slide
Top PRC1 moves
all tension released
PRC1 displacement
all tension released
Microtubule sliding
E
Density of PRC1 (molecules/μm)
Density of attached KIF4A-PRC1 complexes (molecules/μm)
Parameter set 1
Parameter set 2
Parameter set 3
Parameter set 4
F
Overlap length (μm)
Time (minutes)

Figure S5. Simulation details, related to Figure 6.

(A) Components of the computational model. 1) KIF4A and PRC1 can bind to a microtubule from solution if they are within a distance (d_{bind}) with constant rates (k_{on}) and unbind with force-dependent rates (k_{off}). PRC1 and KIF4A bind to different sites on the microtubule, spaced every 8nm, that can only accommodate one molecule. The PRC1 molecules are composed of two identical heads. If one of the PRC1 heads is bound to a microtubule, the other head can bind to a second microtubule within a distance (d_{bind}) with rate k_{on}. 2) KIF4A can attach to PRC1 from solution with rate k_{as}, or from adjacent sites on the microtubule with rate k_{am}, and detach with a force-dependent rate k_d. 3) KIF4A molecules that are attached to PRC1 can bind to microtubules as they do from solution. 4) KIF4A molecules that are attached to PRC1 molecules and bound to a microtubule can either detach from PRC1 or unbind from the microtubule with force-dependent rates (k_{off}, k_d). 5) Both PRC1 and KIF4A cannot move to an occupied binding site, and do not step out of the lattice at either ends. 6) PRC1 heads diffuse on the lattice with hopping rates (h_+, h_-), which depend on the tension in the linker to the other PRC1 head, and eventual KIF4A partner. 7) KIF4A moves towards the plus end with a rate (h_+), affected by the force that drags the eventual PRC1 partner. **(B)** Equivalence of different configurations of Hookean springs as described in the Methods. **(C)** Symbols used in the Methods: *m* the motor, *a* the PRC1 head that is attached to the same microtubule as the motor, and *b* the PRC1 head attached to the opposite microtubule. (**D**) Scheme illustrating the various consequences of KIF4A stepping, eventually either leading to PRC1 'slipping' on both microtubules or to microtubule sliding. **(E)** Equilibrium characteristics of simulation with two microtubules of length 5 μm, 200 PRC1 molecules, and KIF4A molecules varying from 0 to 200, for parameters indicated in Table 1. Dots represent individual simulations placed as a function of the density of PRC1-KIF4A complexes. Note that a density of 125 PRC1/μm corresponds to full compaction. **(F)** Shortening of overlaps in different simulations containing two microtubules of length 5 μm, 100 KIF4A molecules and a PRC1 molecules varying from 100 to 600, with parameter set 1 (Table S1). Time of the graph starts 3 minutes after KIF4A addition, when overlap compaction is reached, as in Figure 6H.

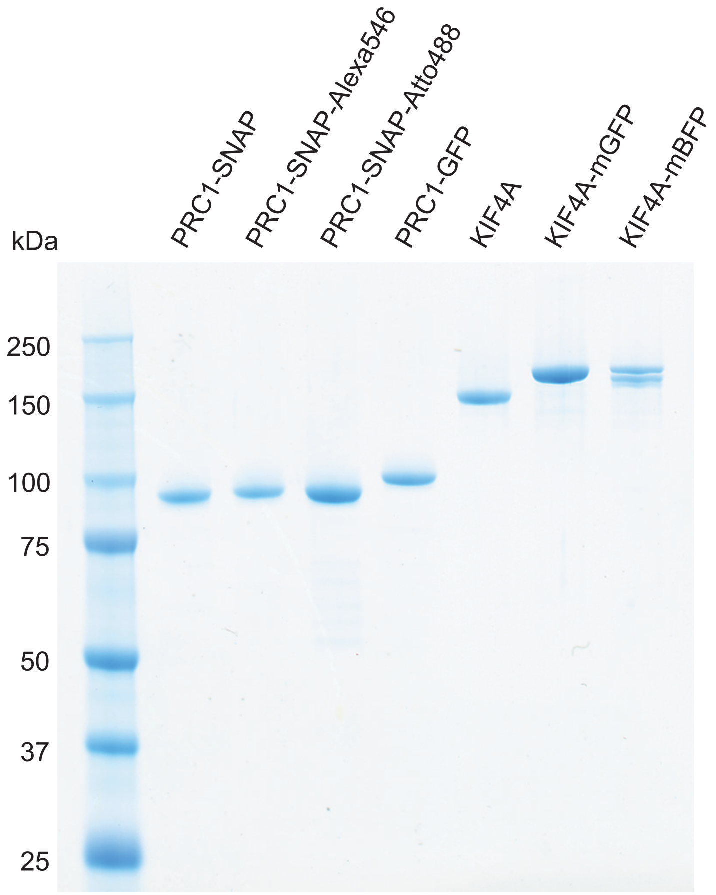

Figure S6. Purified proteins used in this study, related to Figure 1.
Coomassie-stained SDS gel, proteins as indicated.

	Symbol (units)	Description	Set 1	Set 2	Set 3[a]	Set 4
**PRC1**	k_{on} (s^{-1})	Binding rate	0.01	-	-	-
	d_b (μm)	Binding range	0.05	-	-	-
	k_{off} (s^{-1})	Unbinding rate	0.001	-	-	-
	f_u (pN)	Unbinding force	6	-	-	-
	D ($\mu m^2/s$)	Diffusion rate	0.1	-	-	-
**KIF4A**	k_{on} (s^{-1})	Binding rate	0.1	1	0.1	0.1
	d_b (μm)	Binding range	0.05	-	-	-
	k_{off} (s^{-1})	Unbinding rate	0.1	1	0.1	0.1
	f_u (pN)	Unbinding force	6	-	-	-
	v_0 ($\mu m/s$)	Unloaded speed	0.8	-	-	-
	f_s (pN)	Stall force	6	6	6	1
**Interactions**	k_{as} (s^{-1} molecule^{-1})	Attachment rate (solution)	5×10^{-4}	5×10^{-3}	5×10^{-4}	5×10^{-4}
	k_{am} (s^{-1})	Attachment rate (lattice)	1	10	1	1
	k_{off} (s^{-1})	Detachment rate	0.1	1	0.1	0.1
	f_d (pN)	Detachment force	1	-	-	-
	k ($pN/\mu m$)	Linkers stiffness	100	-	-	-

Table S1. Parameters of simulations in Figures 6E, F, S5E, related to STAR Methods.

[a] In parameter set 3, when KIF4A tries to step beyond the plus end, it unbinds from the microtubule and is released to the unbound pool.
