# Supplementary figures and images for: Self-Organization of Minimal Anaphase Spindle Midzone Bundles
Source: Curr Biol. 2019 Jul 8;29(13):2120–2130.e7. doi: 10.1016/j.cub.2019.05.049 (PMC6616649; doi:10.1016/j.cub.2019.05.049)

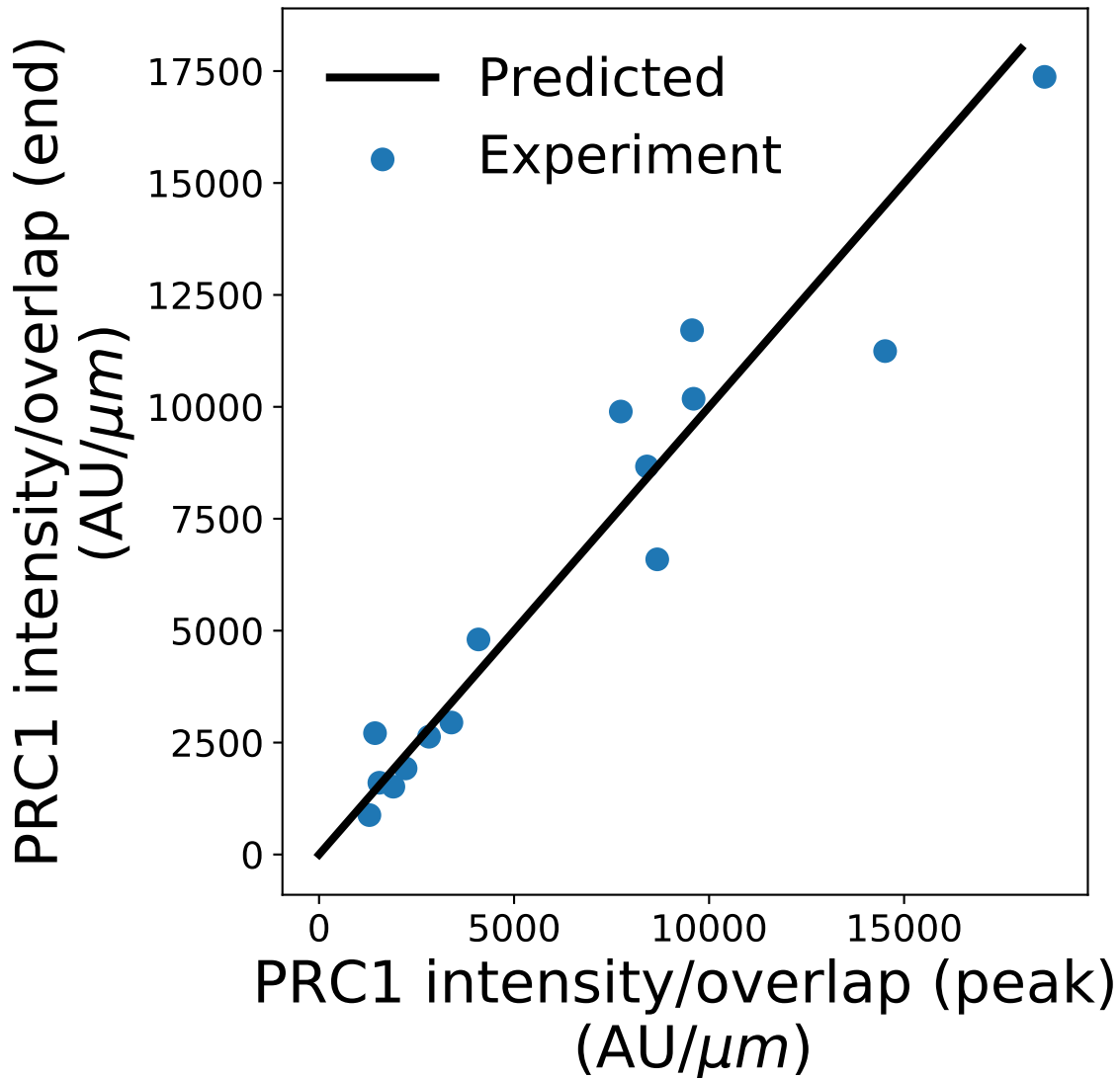

Supplement: Data S1. Simulation Files, Related to STAR Methods — This ZIP contains the source code of Cytosim used for this study, and all the configuration files needed to reproduce the simulation figures. Please, refer to the README file included in the package. [file mmc8.zip › Hannabuss2019_simulation_files_fixed/analysis/figureJ/fig/fig_j.pdf]

Density of PRC1  
(molecules/ $\mu\text{m}$ )

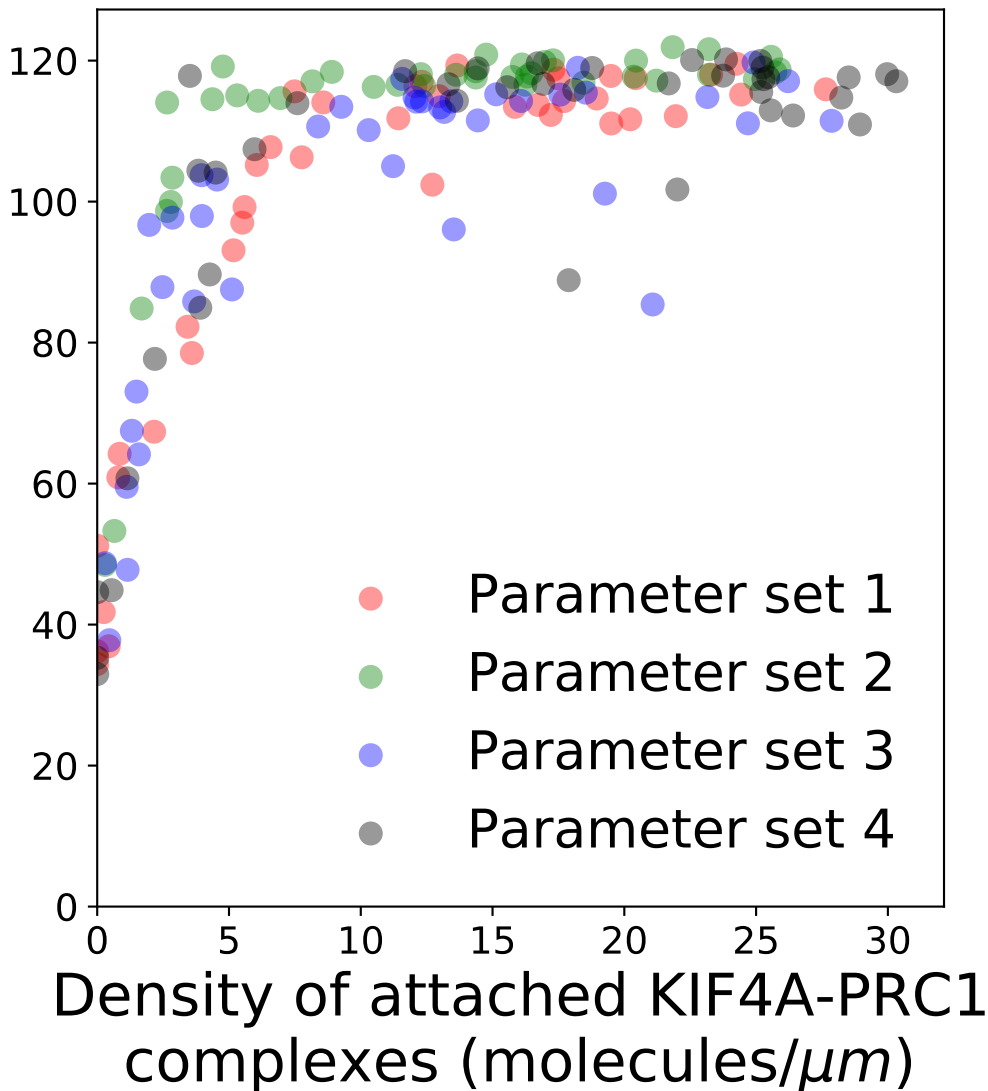

Supplement: Data S1. Simulation Files, Related to STAR Methods — This ZIP contains the source code of Cytosim used for this study, and all the configuration files needed to reproduce the simulation figures. Please, refer to the README file included in the package. [file mmc8.zip › Hannabuss2019_simulation_files_fixed/analysis/figureEF_SE/fig/fig_Se.pdf]

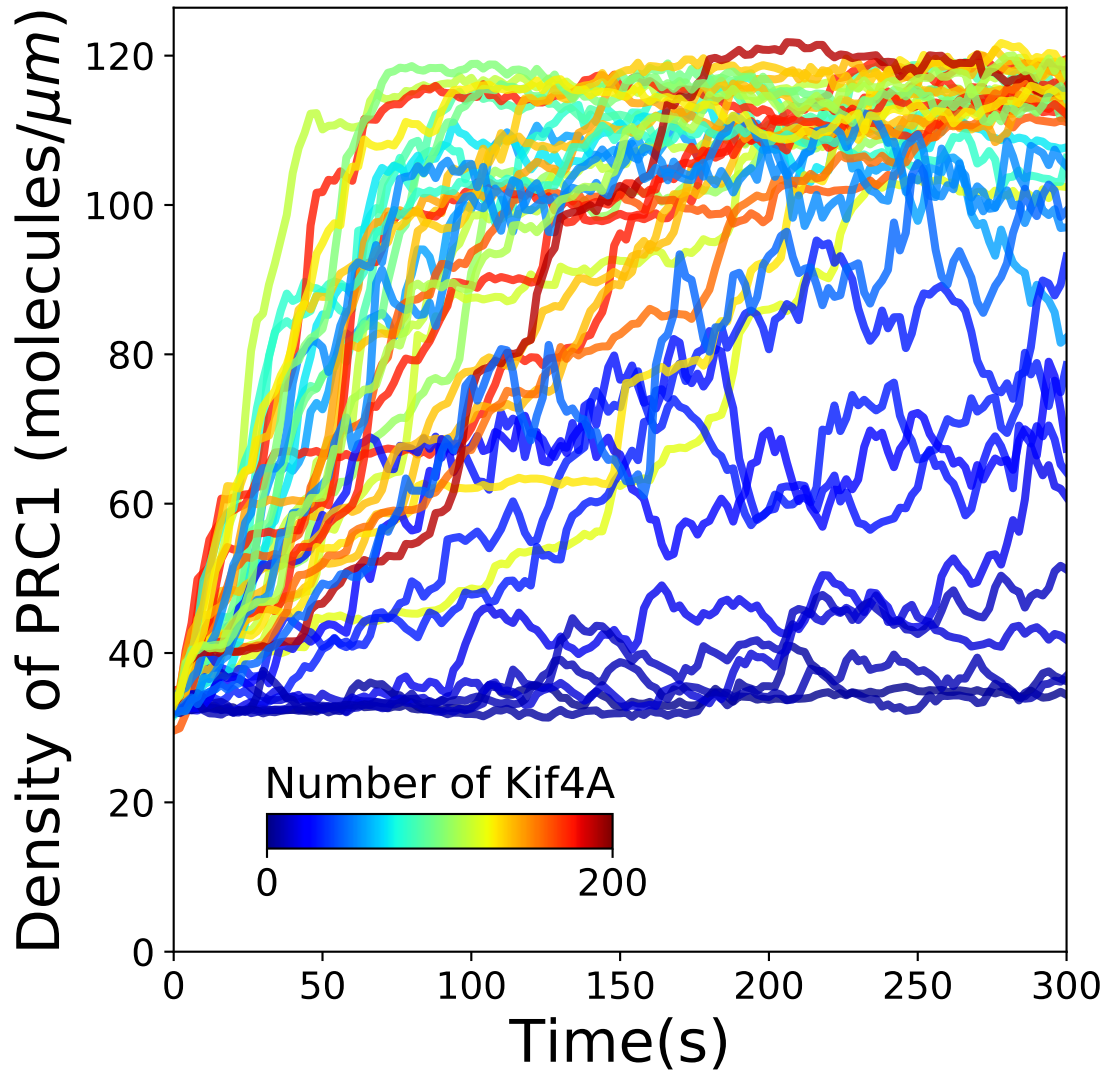

Supplement: Data S1. Simulation Files, Related to STAR Methods — This ZIP contains the source code of Cytosim used for this study, and all the configuration files needed to reproduce the simulation figures. Please, refer to the README file included in the package. [file mmc8.zip › Hannabuss2019_simulation_files_fixed/analysis/figureEF_SE/fig/fig_f.pdf]

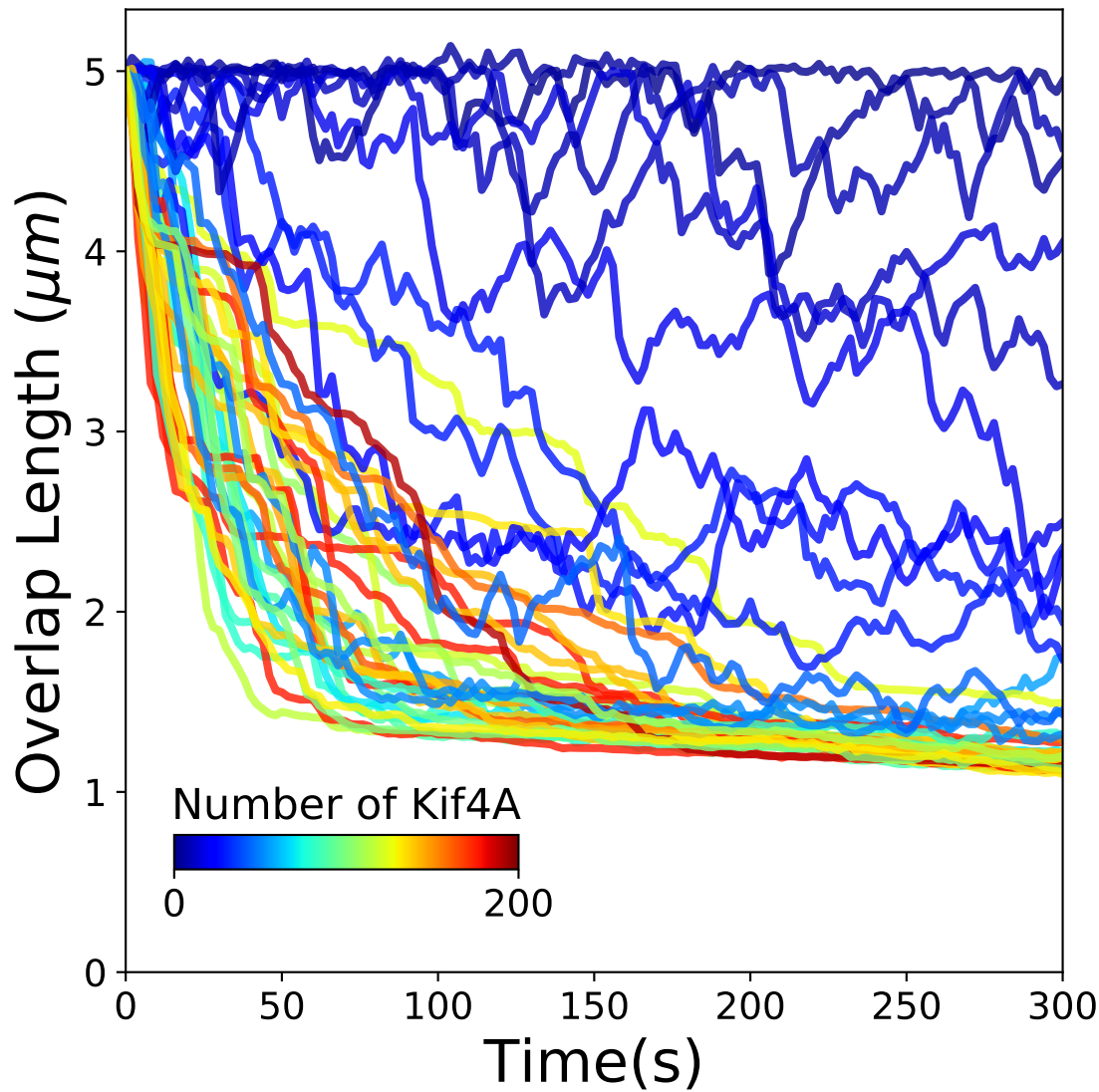

Supplement: Data S1. Simulation Files, Related to STAR Methods — This ZIP contains the source code of Cytosim used for this study, and all the configuration files needed to reproduce the simulation figures. Please, refer to the README file included in the package. [file mmc8.zip › Hannabuss2019_simulation_files_fixed/analysis/figureEF_SE/fig/fig_e.pdf]

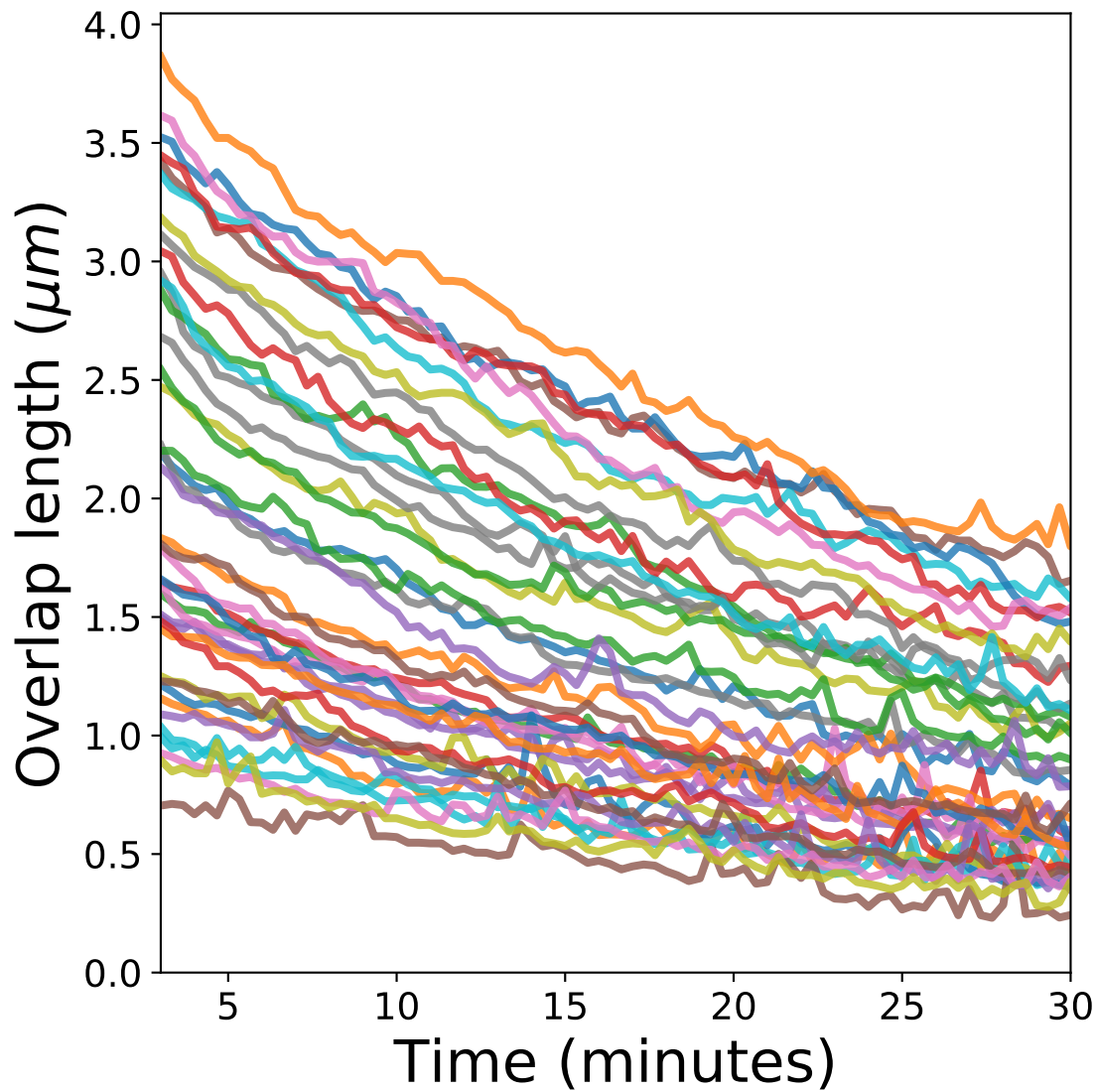

Supplement: Data S1. Simulation Files, Related to STAR Methods — This ZIP contains the source code of Cytosim used for this study, and all the configuration files needed to reproduce the simulation figures. Please, refer to the README file included in the package. [file mmc8.zip › Hannabuss2019_simulation_files_fixed/analysis/figureHI_SF/fig/fig_sf.pdf]

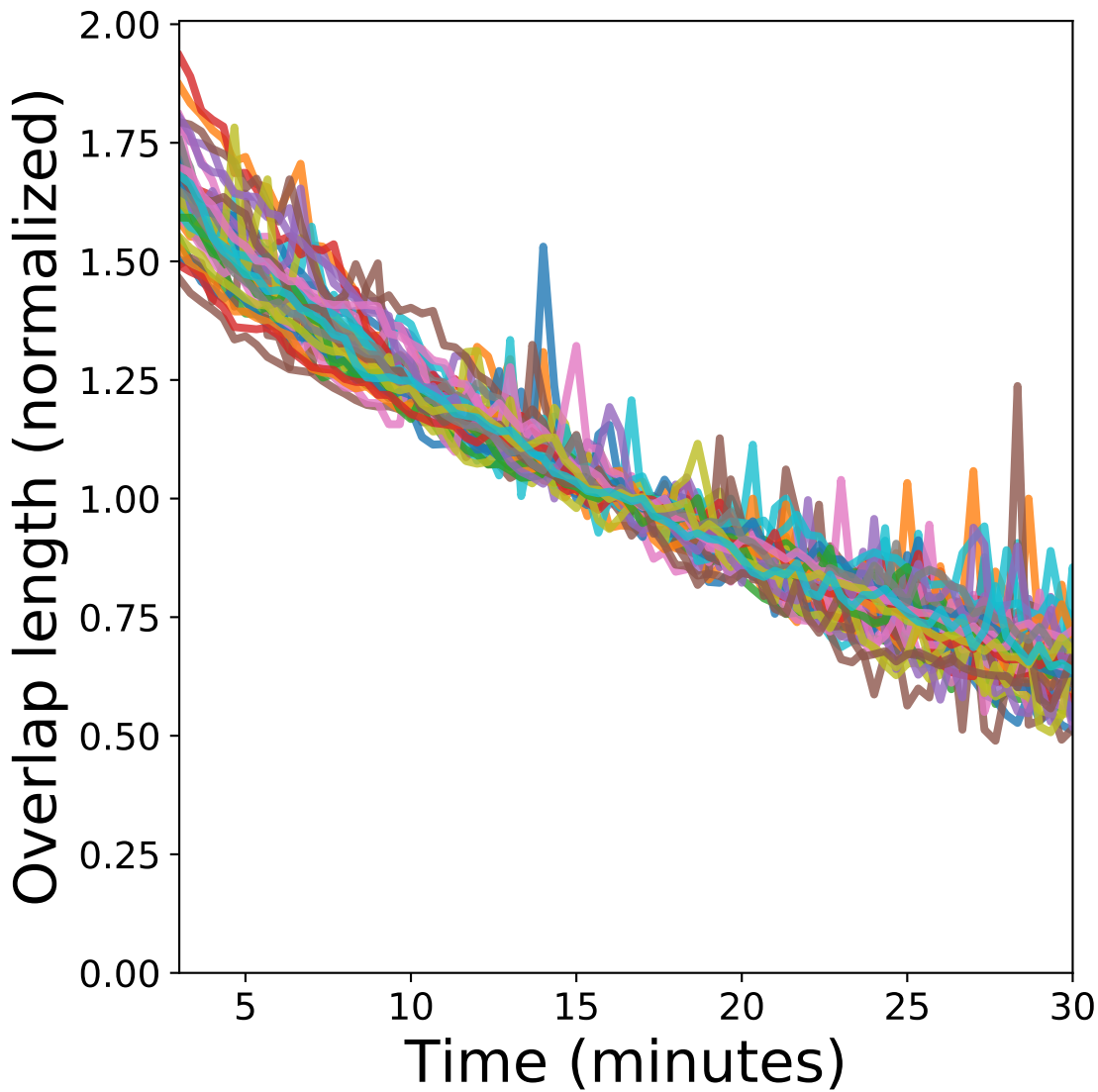

Supplement: Data S1. Simulation Files, Related to STAR Methods — This ZIP contains the source code of Cytosim used for this study, and all the configuration files needed to reproduce the simulation figures. Please, refer to the README file included in the package. [file mmc8.zip › Hannabuss2019_simulation_files_fixed/analysis/figureHI_SF/fig/fig_i.pdf]

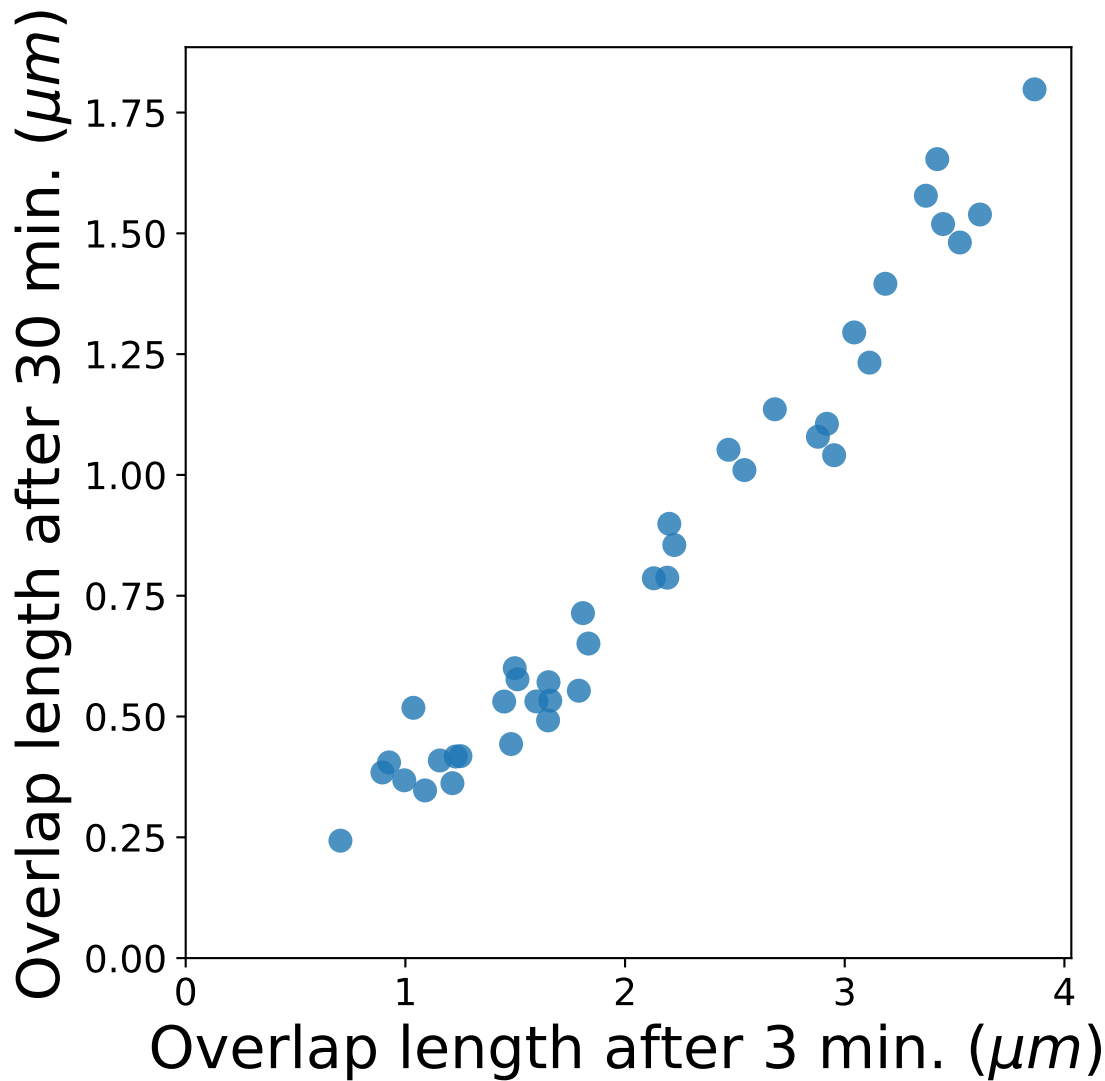

Supplement: Data S1. Simulation Files, Related to STAR Methods — This ZIP contains the source code of Cytosim used for this study, and all the configuration files needed to reproduce the simulation figures. Please, refer to the README file included in the package. [file mmc8.zip › Hannabuss2019_simulation_files_fixed/analysis/figureHI_SF/fig/fig_h.pdf]

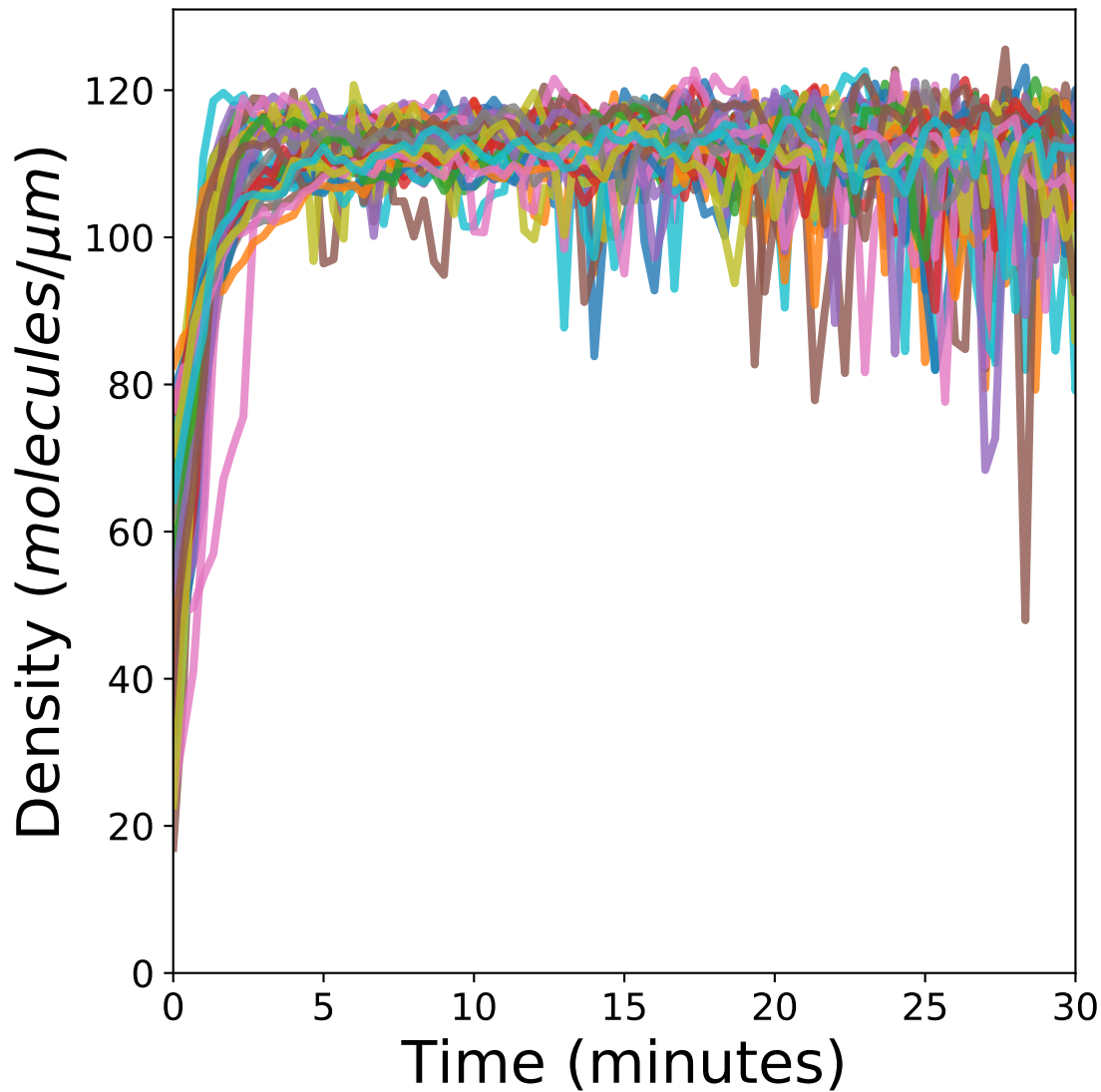

Supplement: Data S1. Simulation Files, Related to STAR Methods — This ZIP contains the source code of Cytosim used for this study, and all the configuration files needed to reproduce the simulation figures. Please, refer to the README file included in the package. [file mmc8.zip › Hannabuss2019_simulation_files_fixed/analysis/figureHI_SF/fig/fig_supp4.pdf]

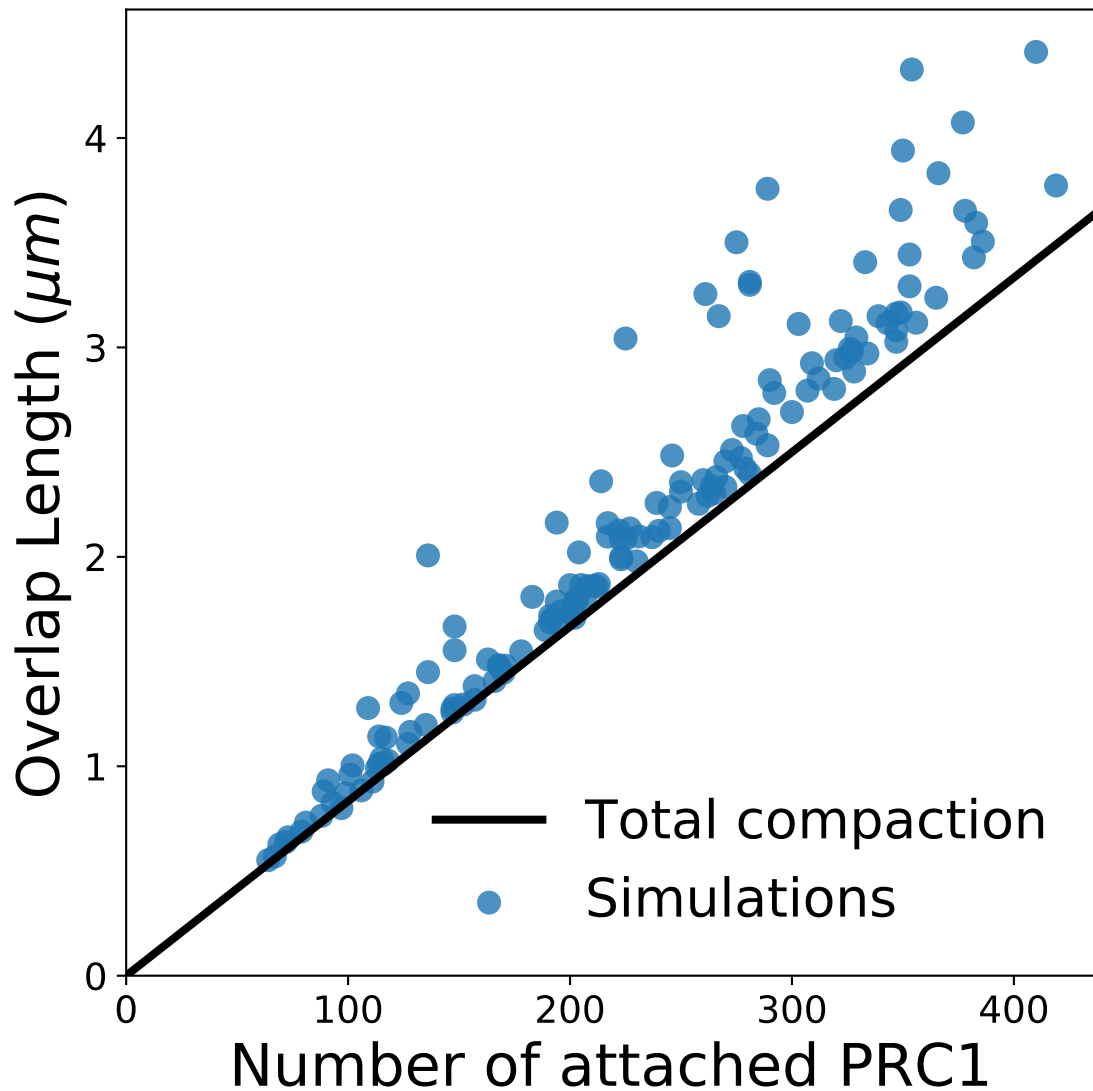

Supplement: Data S1. Simulation Files, Related to STAR Methods — This ZIP contains the source code of Cytosim used for this study, and all the configuration files needed to reproduce the simulation figures. Please, refer to the README file included in the package. [file mmc8.zip › Hannabuss2019_simulation_files_fixed/analysis/figureG/fig/fig_G.pdf]
